# Protolysis Reaction on Pyrophyllite Surface Molecular Models: A DFT Study

**DOI:** 10.3390/molecules30234530

**Published:** 2025-11-24

**Authors:** María Bentabol, Carlos Pérez del Valle, Alfonso Hernández-Laguna, F. Javier Huertas

**Affiliations:** 1 Departament of Inorganic Chemistry, Crystallography and Mineralogy, Facultad de Ciencias, Universidad de Málaga, Campus de Teatinos, 29071 Málaga, Spain; 2 Department of Molecular Chemistry, Université Grenoble Alpes, F-38058 Grenoble, France; carlos.perez@univ-grenoble-alpes.fr; 3 Instituto Andaluz de Ciencias de la Tierra (IACT-CSIC), Consejo Superior de Investigaciones Científicas, Armilla, 18100 Granada, Spain; javier.huertas@csic.es

**Keywords:** pyrophyllite, molecular surface models, DFT, protolysis reaction models, acid dissolution mechanism, surface reactivity

## Abstract

Understanding the mechanisms of mineral dissolution at the atomic scale is crucial for interpreting geochemical processes in soils and sediments, particularly those involving clay minerals. This study addresses the dissolution of pyrophyllite, a model dioctahedral phyllosilicate, under acidic conditions by employing Density Functional Theory (DFT) to simulate protolysis reactions at four distinct edge surfaces ({100}, {010}, {110}, and {130}). Molecular cluster models were constructed for each edge, and the interactions of protons and hydronium ions with various oxygen sites were systematically analyzed. The results demonstrate that bridge oxygens, especially those coordinated to one silicon and two aluminum atoms, are the most reactive sites, undergoing significant bond breaking and structural distortion upon protonation, while hydroxyl groups mainly accommodate structural changes without initiating dissolution. The {110} edge was found to be the least reactive, whereas the {100}, {010}, and {130} edges exhibited the highest reactivity. Hydronium ions produced similar or greater structural changes compared to protons, with water molecules forming hydrogen bonds with the resulting structures. These findings confirm that protonation of bridge oxygens is the rate-limiting step in phyllosilicate dissolution, and that octahedral cations are released preferentially over tetrahedral ones. These findings are consistent with the conclusions drawn from the dissolution experiments. This study provides atomistic information on the dissolution mechanisms of clay minerals at a scale that exceeds the capabilities of dissolution experiments, emphasizing the importance of edge reactivity relative to extensive basal surfaces and the role of water in proton transfer and facilitating protolysis reactions.

## 1. Introduction

Interstitial fluids interact with minerals in sediments, undergoing adsorption, dissolution, and precipitation reactions at the mineral/solution interface. The structure of this region and its physical–chemical conditions (temperature, pH, ionic strength, concentration of organic and inorganic ions, complexing agents, redox potential, etc.) determine mineral evolution. Clay minerals play a key role in the transport and retention of solutes, nutrients, and contaminants in soils and sediments, due to their small particle size, high specific surface area, and capacity to adsorb ions and molecules. The decrease in the dissolution rate of silicates with increasing pH is commonly observed under experimental and environmental conditions. Dissolution and adsorption experiments on clay minerals have shown that the reactivity of the surface changes from one face to another [1,2,3,4,5,6,7]. As a function of the pH, the surface atomic groups undergo protonation or deprotonation reactions. Therefore, dissolution reactions in acidic conditions involve protonation/deprotonation reactions, which trigger the release of structural cations [6,8,9]; the dissolution rate close to neutral pH is the lowest, varying as a function of the nature of the mineral, and as a function of the isomorphic substitutions in consequence. It has been suggested that the rate-limiting step in the dissolution of aluminosilicates was the breaking of bonds to the bridge oxygens, Al–O–Si (e.g., [2,6,10,11]). Nevertheless, experimental methods cannot be used to fully identify some individual steps of the dissolution reaction mechanism, such as the surface groups where protons were specifically adsorbed to decompose the structure through a protolysis reaction. Xio and Lasaga [12], in a pioneering ab initio study, identified the proton attack on bridge oxygens as the step controlling the dissolution reaction.

Hendricks [13] deduced the development of surface charge on the edge face of crystalline silicates, and Schofield and Samson [14] modeled the clay mineral edge facets and their capacity to exchange protons and hydroxyls. Later, White and Zelazny [15] reported an exhaustive crystallochemical analysis of the edge structure of dioctahedral phyllosilicates. Bleam et al. [16] concluded that pyrophyllite {100} or {130} edge faces seemed to be more reactive than {110} or {010} edge faces, which may explain the crystal shape in natural specimens being dominated by {010} and {110} faces. Therefore, the analysis of the structure and facets of phyllosilicates reveals surface reactive sites with oxygen atoms exhibiting differential characteristics. Limiting to dioctahedral minerals, siloxanic oxygen bonds (Si–O–Si) form the basal surface of the tetrahedral sheet. Bridge oxygens connect the tetrahedral and octahedral sheets, bonding a tetrahedral and two octahedral cations (Si–O < Al_2_). Finally, the oxygen of the hydroxyl groups is shared by two octahedral cations (Al-OH-Al). At the edge faces, these oxygens may have dangling bonds that relax by protonation and/or deprotonation, forming silanol (Si–OH) and aluminol (Al-OH) groups, and adsorption of water molecules (Al–OH_2_) [17].

Besides experimental procedures, computational methods can also be used to study the structure, properties, reactivity, and mechanisms at the mineral/solution interface, serving as an additional method to complement experimental inferences and theories. Concerning the dissolution processes of phyllosilicates, computational studies enable us to examine reaction steps that are not accessible through experimental methods. Different types of theoretical surface models are often employed in these studies. Some models involve two main approaches: (i) the crystallographic approach, and (ii) the local approach. The first approach treats the surface as a whole by selecting a crystallographic plane, creating a vacuum within the crystal structure, and applying periodic boundary conditions. In contrast, the local approach also selects a crystallographic plane but models only the local structure of that plane as a molecular system. Density Functional Theory (DFT) is mainly used in the physical chemistry of crystals for both surface models [18,19,20]. Both approaches involve different modeling choices and electron DFT parameters. The crystallographic approach must account for the size of the slab layers, the number of layers optimized in the surface and adsorption model, the vacuum height, the cell or supercell, etc., the exchange and correlation functional, the basis set size, the k-point sampling, and the cutoff parameters, among other factors. The local approach also needs to consider the size of the local site, the cluster size, the closing of dangling bonds, the basis set size, the exchange and correlation basis set, solvent environment, etc. (e.g., [21,22]). These methods help explain many surface processes at the microscopic level through local atomic interactions. These studies often provide qualitative and sometimes quantitative insights into the physicochemical processes at mineral interfaces. The crystallographic approach has been applied with the DFT method to study water adsorption on the surface and in the interlayer space of Na- [23] and Ca-Montmorillonite and kaolinite [24], yielding clear results. Local molecular systems of moderate size, combined with DFT methods, have been applied to clay minerals (e.g., [25,26]). Sainz et al. [27] reported DFT research on the structure of dioctahedral TOT phyllosilicates (O and T refer to octahedral and tetrahedral sheets, respectively) with high, medium, and low interlayer charges. Molecular cluster models have been successfully used in studies of acid-treated montmorillonite, showing that isomorphic substitutions of Al^3+^ by Mg^2+^ in the octahedral sheet led to effective protonation of test molecules, more so than substitutions of Si^4+^ by Al^3+^ in the tetrahedral sheet [28]. Therefore, theoretical surface models of phyllosilicates should provide a comprehensive understanding of protolysis reactions, with results that can help interpret experimental findings, propose reaction mechanisms, and support broader extrapolations.

**Figure 1:**
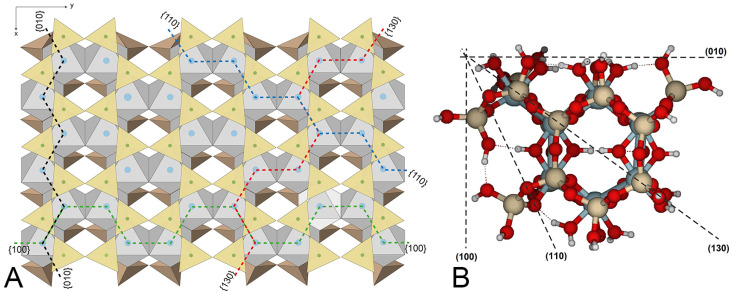
(**A**) Idealized view of the pyrophyllite structure on the ab plane, based on Bleam et al. [16]. Octahedral distortion was not included. Dotted lines depict periodic bond chains (PBC) for the four edge faces studied, centered on octahedral cations (Al, blue dots). Note the similarity between the {100} and {130} faces, as well as the {010} and {110} faces. (**B**) Top view of the pyrophyllite cluster model from the [001] perspective. Dashed lines represent the Miller indices of lattice planes parallel to the edge faces. Red spheres: oxygen; white spheres: hydrogen; beige spheres: silicon; and blue-grey spheres: aluminum. This labeling convention is used throughout the article. The figure design follows the guidelines outlined by Lavikainen et al. [30].

Pyrophyllite is a 2:1 dioctahedral phyllosilicate with no isomorphic substitutions and does not possess any structural charge (Figure 1A). Thus, it is the simplest model for a large variety of clay mineral studies. There are many studies related to the basal surfaces of these minerals, although the basal plane is much less reactive than the edges of the clay particles [29]. Few studies have modeled the edge faces of phyllosilicates.

Churakov [31] calculated the composition and relative stability of the edge faces of pyrophyllite and later provided results on the proton exchange process on the hydrated lateral facets. He found that the Si–O–Al groups had the highest proton affinity on {110}, {100}, and {130} edges. However, in the {010} edge, the proton affinity was associated with the Al–OH groups.

Systematic quantum mechanical calculations of pyrophyllite edge surfaces revealed that, although the basal {001} surface is hydrophobic, the edge surfaces display hydrophilic properties due to hydroxyl groups and dangling atoms [32]. Ulian and Valdrè [33] thoroughly characterized pyrophyllite thermochemical and thermo-physical properties using DFT, providing data on heat capacity and thermal expansion coefficients across a broad temperature range (0–900 K).

The most recent studies on molecular dynamics of dissolution at the {110} edge facets of pyrophyllite revealed that dissolution in pure water is a complex process with multiple steps, characterized by a series of simultaneous direct and reversible elementary reactions [34]. These reactions result in a gradual reduction in surface site density, ultimately leading to the release of silanol and aluminol complexes into the solution.

It is experimentally clear [35] that protons are involved in the dissolution mechanism of silicates; however, the precise mechanism is not yet clearly understood. Therefore, this study aims to enhance our understanding of the dissolution mechanisms of dioctahedral phyllosilicates in acidic conditions by providing atomistic evidence, given their importance in geological and environmental processes. To achieve this, DFT calculations will be used to model the protolysis reaction at the edge faces. Molecular models for four edge faces—{100}, {010}, {110}, and {130}—will be developed. The reactivity of each face during the protolysis process will be assessed based on its Proton Reaction Energy (PRE) and the distortion of its structure after protonation.

## 2. Results

**Figure 2:**
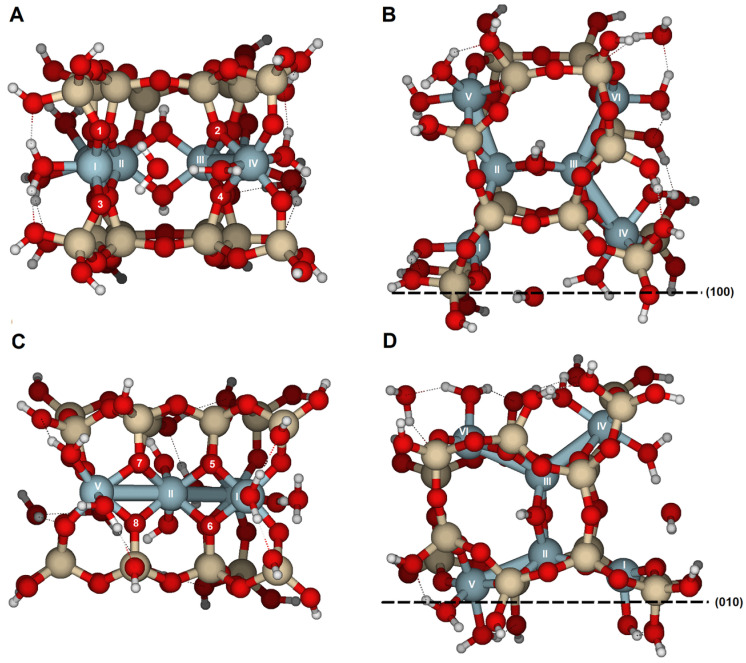
Cluster model (Si_16_Al_6_O_56_H_30_ · 2H_2_O) used to examine the protolysis reaction at the {100} and {010} edge facets in pyrophyllite. Al^3+^ ions labeled I to IV are the most external; there are two less obvious Al^3+^ ions in the middle of the cluster, labeled V and VI. (**A**) View parallel to (100), and (**B**) top view of the (100) face looking toward the [001] direction. (**C**) View parallel to the (010) lattice plane, and (**D**) top-down view of the (010) edge face facing the [001] direction. Numbers label potential reactive O atoms on each edge face, and Roman numerals identify Al atoms. Dashed lines with Miller indices indicate the lattice planes, which are parallel to the cleaved edge faces. This convention is used throughout the document.

**Figure 3:**
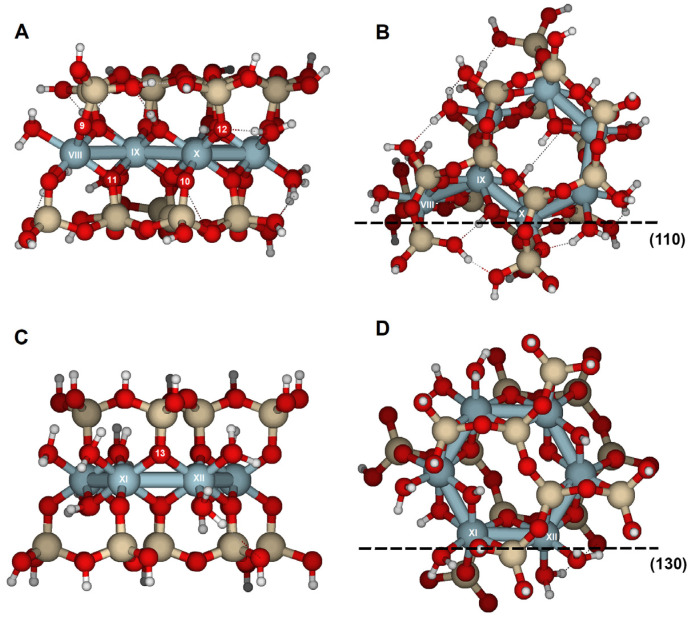
Cluster model of pyrophyllite edges. (**A**) Cluster model (Si_17_Al_7_O_62_H_35_ · H_2_O), aligned with the {110} lattice plane; and (**B**) top-down view of the {110} edge face along the [001] direction. (**C**) cluster model (Si_14_Al_6_O_56_H_30_ · 2H_2_O), aligned with the {130} lattice plane; and (**D**) top-down view of the {130} edge face along the [001] direction. Numbers label potential reactive O atoms and Roman numerals identify Al atoms. Dashed lines with Miller indices indicate the lattice planes, which are parallel to the cleaved edge faces.

The initial molecular model’s geometry was based on an ideal pyrophyllite crystal structure (Figure 1), which is consistent with another previously published pyrophyllite model [34]. From this structure, molecular clusters of different surfaces were separated, and dangling bonds were closed with hydrogen atoms or water molecules. These molecular cluster models were optimized, resulting in the structures shown in Figure 1B, Figure 2 and Figure 3. As a result of isolating the molecular structure of the periodic crystal, a slight slip of the octahedral sheet occurred after optimization, causing the tetrahedral sheets to shift relative to each other. This slip may result from the different approximations used during modeling and calculations. Using these previous molecular structures as reactants, protons or hydroniums were added near the designated reactive atoms, followed by full optimization. The resulting structures were identified as reaction products. The root mean square (RMS) of bond length differences between reactants and products serves as a measure of the structural distortion caused by protonation and protolysis. These values reflect the initial stages of the dissolution process. Additionally, specific bond lengths are highlighted to emphasize the most reactive bonds.

Transition states between the reactants and the products were searched, but the results were unsatisfactory. Thus, protolysis probably occurred directly, without passing through an intermediate state. In all the reactions modeled, no energy barrier was found, which is consistent with the result of molecular dynamics [34].

### 2.1. {100} Edge Surface

The model was created on one of the edge faces, such as {100}, which was cut through the periodic structure along the indicated plane (Figure 2). Referring to Figure 1 and the dashed line following the Al^3+^ in the octahedral sheet, six Al^3+^ ions (labeled with Roman numerals in Figure 2A,B) were selected from two adjacent octahedral cavities. The OH groups are *trans*-disposed between Al(II) and Al(III). As a result, two tetrahedral rings are positioned above and below, with half of the two broken tetrahedral rings exposed parallel to the {100} face. Dangling bonds, such as Si–O or Al–O, were closed with either H or H_2_O, respectively. Two H_2_O molecules were placed near Al(I) and Al(IV) to maintain octahedral coordination. The surface model was fully optimized.

**Figure 4:**
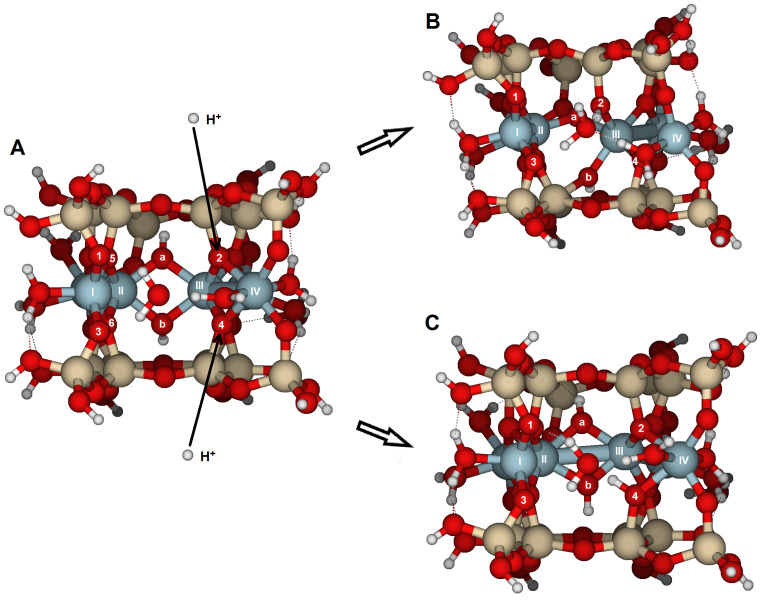
Structure of edge facets in pyrophyllite parallel to the {100} lattice planes: (**A**) the most stable optimized edge structure (**A**) before protonation; (**B**) after attack with a proton across O2; and (**C**) after protonation across O4. Oxygen atoms labeled as O*a* and O*b* in the figure are located at depths where protonation is not feasible. Numbers label potential reactive O atoms, and Roman numerals identify Al atoms.

Several potential attack sites for H^+^ are shown in Figure 2A,B, from O1 to O4. Atomic distances for the initial and protonated optimized configurations are listed in Table S1 and RMS and PRE are listed in Table 1. Reaction of H^+^ at either O1 or O3 causes only limited reorganization of the model. The RMS values for attacks on O1 (0.066 Å) and O3 (0.052 Å) suggest minor geometric distortions. However, when H^+^ attacks O2 or O4 (Figure 4), significant reorganization occurs, with RMS values (0.420 and 0.265 Å, respectively) larger than those for O1 and O3. The processes of bond breaking and bond forming are discussed in detail.

(i) O1 and O3 are bridge aluminol oxygens (Si–O–Al) located on the outer surface with two free lone electron pairs, so they act as Brønsted base sites, with a high PRE. The proton attack causes limited global geometric distortions and low RMS values. Protonation of the oxygens increases Al(I)-O1 and Al(I)-O3 bond distances by 0.16 and 0.13 Å, respectively. Therefore, the Brønsted basic sites do not appear to be critical for the initial steps of the dissolution reaction, although all protonations somewhat influence the overall dissolution process.

(ii) O2 is a bridge oxygen (Si–O < Al_2_) located inside the cavity, providing increased electron density engaged with the surface and only one lone electron pair available to react with the proton. Protonation triggers significant bond-breaking and bond-forming processes on the surface (Figure 4), leading to the highest RMS value (0.420 Å). There are also two hydroxyl groups [Al(II)–OH–Al(III)] (O atoms of the OH groups are labeled *a* and *b* in Table S1). The Al(II)–O*b* bond length increased from 1.957 to 2.874 Å, which may indicate a break in the Al(II)–O*b* bond. Additionally, O*b* formed a new bond with one of the Si atoms in the lower tetrahedral sheet of the model (Si–O*b*–A(III). Furthermore, the Al(IV)–O2 bond also dissociates as its length increases from 1.986 to 3.337 Å, reducing the coordination of Al(IV) and increasing the distance between Al(III) and Al(IV) (3.033 to 3.489 Å). Despite these bond-breaking and bond-forming processes, this was the most stable structure with the highest PRE (22121030 kJ/mol, Table 1). The PRE associated with this configuration (Figure 4B) is 36 kJ/mol higher than the optimized structure protonated at O3. This reaction on the bridge oxygens can be considered one of the most critical steps in the dissolution process. These PREs are in qualitative agreement with the relative values reported in ref. [31] for low- and high-water coverages.

(iii) Nevertheless, if the proton reacts with O4, the most significant change is the dissociation of the Al(III)-O4 bond, which extends from 1.935 to 2.952 Å (Table S1, Figure 4C). A similar bond-breaking and bond-forming process occurs as in the previous O2 protonation. The coordination of water to Al(IV) remains largely unchanged. The Al(II)–(OH)_2_–Al(III) group stays intact, which distinguishes it from the O2 attack. The PRE was −971 kJ/mol. The moderate RMS value of 0.265 Å suggests limited reorganization.

For the initial protonation of the {100} surface, the overall RMS (for a series or a face, the overall RMS value was calculated using all the corresponding optimized configurations) and average PRE across the four reactive oxygens are 0.252 Å and −993 kJ/mol, respectively. These variations in the model are attributable to the strength or weakness of the atom coordination. Strong coordination allows a proton to cause significant structural changes; meanwhile, weak coordination, on the other hand, may result in weaker structural changes. These features might be due to limitations in the model. However, it is important to recognize that similar imperfections also exist on real surfaces exposed to environmental conditions [16]. Therefore, the initial steps of the dissolution process can be viewed as a series of elemental reactions—some causing minor distortions, others leading to more significant structural changes that break or form bonds at the surface—all of which contribute to surface dissolution. This multi-step mechanism agrees with the mentioned one in ref. [34].

**Figure 5:**
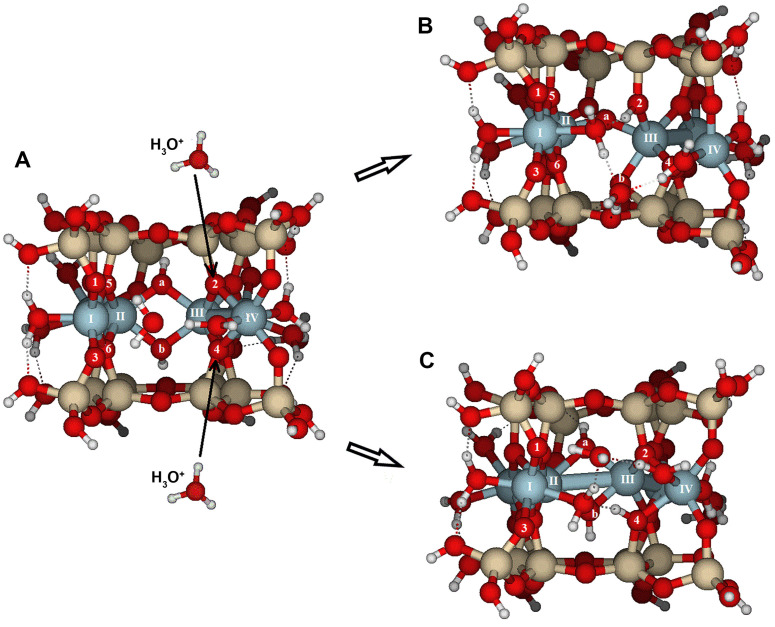
Structure of edge facets in pyrophyllite parallel to the {100} lattice planes: the most stable optimized edge structures (**A**) before protonation, after protonation with H_3_O^+^ (**B**) across O2; and (**C**) across O4. Oxygen atoms labeled O*a* and O*b* in the figure are located at depths. Numbers label potential reactive O atoms, and Roman numerals identify Al atoms.

When the reactants were hydroniums instead of protons, the final configurations showed a high degree of similarity with those of protons, with similar RMS values (overall RMS = 0.252 Å, Table 1, Figure 4 and Figure 5). However, following Equation (2), PREs (∆E_2_, Table 1) were much lower than those of one-proton structures, being even one-third of the previous PREs. The average PRE for the hydronium ions is −363 kJ/mol. These low PREs could result from the partial hydrolysis of the product water molecule originating from the hydronium reactant. The primary effects were carried out by the hydronium at O2, with the proton of H_3_O^+^ transferring to O2 (Figure 5), producing similar bond-breaking and bond-forming effects as the H^+^ on O2 (similar RMS values, same maximum bond-breaking/bond-forming lengths, Figure 4B and Figure 5B). The impact on O4 is very similar to that on O2: a proton was transferred to O4, and the bond-breaking lengths were similar to those in the O2-proton structure. In any case, the proton transfers play an important role at the intermediate critical point (CP) of the potential energy surface (PES) of the dissolution reaction. Possibly, the sites of water molecules coming from the hydronium in the models are limited by the model itself, and in a real system, they would migrate to the hydration sphere, resulting in similar PREs for S–H^+^-hydration-sphere and H_2_O–S–H^+^-hydration-sphere systems (see Equations (1) and (2), Section 4.1).

If a second proton reacts with the protonated structure (either S-H^+^ or H_2_O-S-H^+^), different scenarios can occur (Table 2). The addition of a second proton causes significant distortions in the molecular model. The most notable distortions observed were characterized by high RMS values (around 0.8 Å) and bond lengths increased to around 3.5 Å.

**Figure 6:**
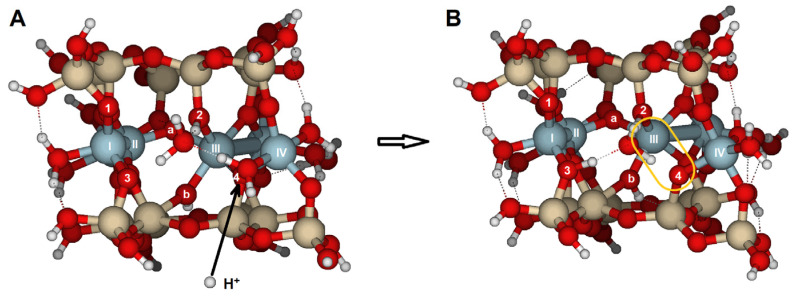
Structure of edge facets in pyrophyllite parallel to the {100} lattice planes: the most stable optimized edge structures (**A**) after a first attack with a proton across O2; and (**B**) after the attack of a second proton across O4. Note that in the final configuration, the proton is attached to O3. Yellow ovals show the elongated Al(III)–O4 bond. Numbers label potential reactive O atoms on each edge face, and Roman numerals identify Al atoms.

**Figure 7:**
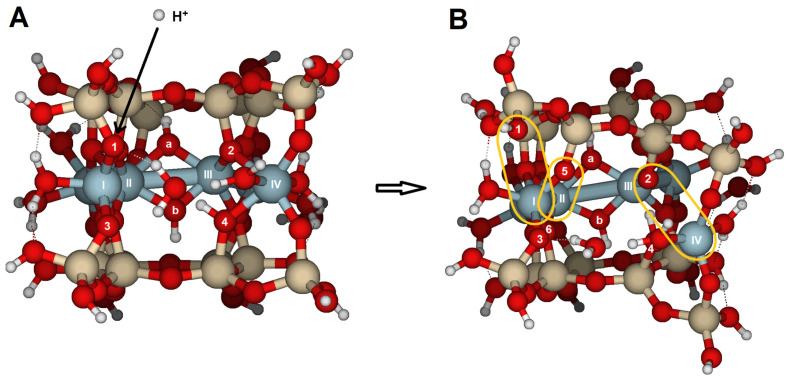
Structure of edge facets in pyrophyllite parallel to the {100} lattice planes: Optimized structure (**A**) after protonation at O4, and (**B**) after a second H^+^ attack across O1. Yellow ovals indicate the elongated bonds after the second attack: Al(I)–O1, Al(II)–O5, and Al(IV)–O2. Numbers label potential reactive O atoms, and Roman numerals identify Al atoms.

(i) The first attack on O1 only causes an increase in the Al(I)–O1 and Al(II)–O5 bond distances, but no significant bond-breaking is observed, indicating minor changes in the RMS geometry. However, after this attack, a second H^+^ reacts with O2, leading to a substantial distortion of the structure, with one of the largest RMS values (0.829 Å). First, the Al(I)–O1 bond breaks, opening the tetrahedral sheet and making it more vulnerable to further attacks by protons and hydrolysis. Similar bond breaking occurs at Al(II)–O5 and Al(III)–O2. These structural breakages proceed to the advanced stages of surface.

(ii) If the initial one-proton attack was carried out on O2, a subsequent H^+^ reaction either on O1 or O3 caused only minor distortions, as indicated by the low RMS values (0.084 and 0.053 Å, respectively). The PREs were approximately 100–200 kJ/mol lower than those in the previous single-proton reaction. However, if the second reaction occurred on O4, it resulted in an RMS of 0.440 Å. After the attack, the H^+^ migrates to O3, forming hydrogen bonds between the water molecule and the protonated O2 and O3, and causing the breaking of the Al(III)–O4 bond (Table S2, Figure 6), which constitutes the most significant structural distortion. Nonetheless, the PREs for the three potential attacks starting on O2 are similar.

(iii) Alternatively, an initial attack on O4 followed by a second attack via O1 caused significant changes in the structure, as indicated by the largest RMS value for the series (0.969 Å, Table 2, Figure 7) and broken bonds. Before protonation, O1 acted as a Brønsted base site with two lone electron pairs, one of which is engaged in a hydrogen bond with the coordination water, while the other pair was available to accept a proton. The observed step sequence was as follows: (1) The Al(I)–O1 bond lengthened from 1.845 to 4.830 Å and broke, leading to one of the steps of the detachment of octahedral and tetrahedral sheets and the formation of a silanol group (Si–O1–H). (2) The Al(II)–O5 bond broke (from 1.938 to 3.743 Å), and (3) the Al(III)···O4 distance increased to 3.411 Å, causing Al(IV) to dissociate from O2 as well (from 2.121 to 3.548 Å). (4) These structural changes led to the detachment of the tetrahedral sheets from the octahedral sheet. (5) Finally, the Al(I) coordination number decreased from six to five. The PRE (∆E_3_ (1H^+^)) reached its maximum at −1004 kJ/mol. The PRE value for the two-proton reaction (∆E_5_ (2H^+^)) was also the highest. When considering only the second proton attack on the {100} face, an overall RMS of 0.513 Å and average PRE of −828 kJ/mol were observed for the complete series. The overall RMS is twice that of a single H^+^ attack, and the PRE is lower than the average PRE for one H^+^ attack. Therefore, the destruction of the structure is more extensive than the first H^+^ attack. Consequently, at low pH, crystal dissolution is expected to be more intense than at higher pH values. Higher temperatures also increase H^+^ collisions on the surface, further enhancing the dissolution of the structure.

In summary, the two-proton attack on the {100} surface breaks multiple bonds. It causes notable structural distortions, which likely result in the detachment of structural sheets, cation loss, and mineral dissolution. Schiemann and Churakov [34] calculated the detachment of both sheets using Molecular Meta-dynamics, employing coordination number collective variables and describing a multi-step reaction mechanism [31,34], as calculated in our study. Globally, the structural changes indicated by the RMS are generally larger than those caused by the one-proton attack.

### 2.2. {010} Edge Surface

In the {010} surface model (Figure 2C,D), there are four options for attacking bridge oxygens, which also cause significant structural distortions, as indicated by the RMS values (0.961–0.248 Å, Table 3 and Table S3). The overall RMS value is 0.605 Å, which is 2.5 times higher than on the {100} surface (0.252 Å).

When one H^+^ reacted with O7, the Al(II)–O7 bond length increased from 1.969 to 3.614 Å (Table S3). The attack on O8 caused milder bond breakage and structural distortions, but in this case, the distortion occurred around Al(V) instead of the central Al(II), with the Al(V)–O8 bond length increasing from 1.954 to 3.181 Å. O8 (PRE = −902 kJ/mol) could be considered the least basic oxygen on this face. The average PRE of the first attack on this surface is −975 kJ/mol, slightly lower than the value for the {100} surface. This event can be seen as one of the initial steps toward detaching the octahedral sheet from the tetrahedral sheet and subsequent protolysis/hydrolysis.

**Figure 8:**
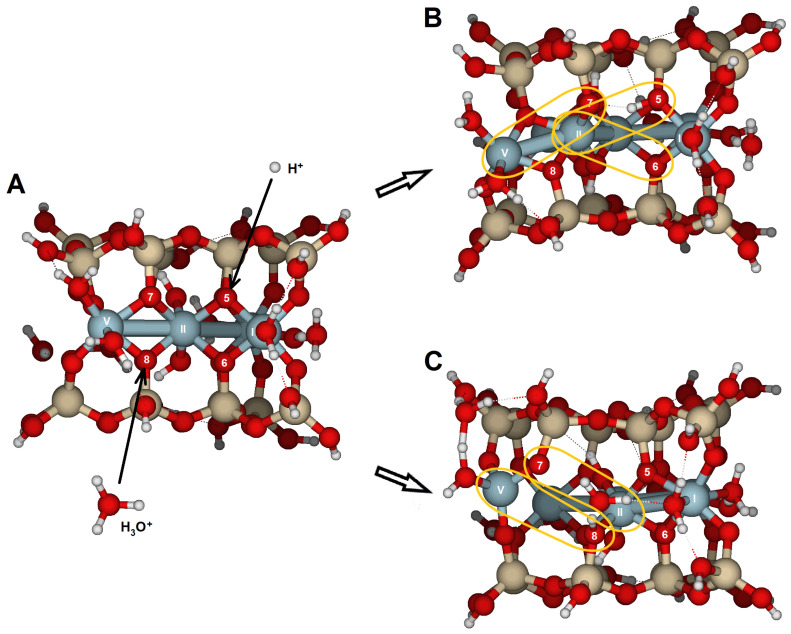
Structure of edge facets in pyrophyllite parallel to the {010} lattice planes: the most stable optimized edge structures (**A**) before protonation; (**B**) after optimization with protonation (H^+^) across O5; and (**C**) when attacked by an H_3_O^+^ ion across O8. Yellow ovals indicate elongated bonds after the attack. Numbers label potential reactive O atoms, and Roman numerals identify Al atoms.

The most significant structural change occurs when the H^+^ attacks O5 (Figure 8). Protonation of O5 breaks the bonds Al(II)–O5, Al(V)–O7, and Al(II)–O6, causing Al–O distances to increase from about 2.0 Å to over 3.3 Å. Additionally, the distance between Al(II) and Al(I) increases by 1.5 Å. This attack thus triggers the dissociation of the octahedral sheet. The RMS (0.961 Å) of this attack is the largest in the series. This protolysis is one of the most structurally damaging events for the {010} surface and plays a key role in the dissolution process. Furthermore, the PRE is −1024 kJ/mol, one of the highest PRE values calculated with these models. These PREs agree with the relative enthalpies of Churakov [31] on the low- and high-water coverages.

When hydronium was the reactant, the distortions resembled those of the proton case, with similar RMS values (overall 0.609 Å, Table S3). The largest RMS in the series was observed for the addition to O8 (Figure 8C). Significant elongations of the Al(V)–O8 and Al(II)–O7 bonds were observed, with the Al(V) coordination decreasing from six to four, preparing the octahedral sheet for separation from the tetrahedral sheet. The water molecule stayed inside the cavity and formed hydrogen bonds with protons. The PREs calculated with Equation (2) are roughly one-third of the PRE for a single proton attack, as was previously seen on the {100} surface.

Additional calculations were performed to assess the impact of a second proton on the structure. If the first proton attached to O6 (Table 4 and Table S4), the Al(II)–O6 bond lengthened from 2.026 to 2.719 Å. There were three possible sites for the second H^+^ to react: (i) if the subsequent H^+^ attacked O5, two bonds broke: Al(II)–O6 (2.719 to 4.029 Å) and Al(I)–O5 (1.930 to 3.886 Å), with an overall RMS of 1.025 Å. (ii) When the second proton reacted with O7, it caused the most significant distortion in the series (RMS = 1.260 Å), mainly due to the enlargement of Al(V)–O7, Al(II)–O5, and Al(I)–O6 bonds. (iii) Conversely, if the second protonation occurred on O8, the perturbation was smaller, resulting in the lowest RMS value among the series (0.591 Å). The overall RMS for the second H^+^ attack was 0.998 Å, higher than the value for the one H^+^ attack (0.605 Å), because the second protonation enhanced distortions. Note the significance of the first H^+^ attack in the overall reaction energies, the Brønsted basicity of the atoms, and the structural changes on the surface.

Despite the structural differences caused by this double attack, the most energetically stable double-protonated model involved the first H^+^ attaching to O8 and the second attack on O5, with the largest PRE of −1056 kJ/mol for the second H^+^ (Table 4). Additionally, the PRE of this group was the highest (average PRE on O8 is −910 kJ/mol for the second H^+^ and −1811 kJ/mol for the two-H^+^-attack). However, the structure becomes more distorted due to the initial protonation and weak coordination of some atoms. As a result, the basicity of certain oxygen atoms may increase because of the distorted structure.

**Figure 9:**
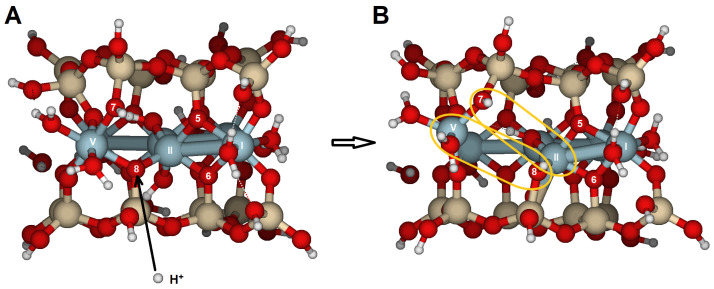
Structure of edge facets in pyrophyllite parallel to the {010} lattice planes: (**A**) the most stable configuration after protonation in O7, and (**B**) after a second H^+^ attack across O8. The yellow ovals show the bond elongation after the second H^+^ attack: Al(V)–O8 and Al(II)–O7. Numbers label potential reactive O atoms, and Roman numerals identify Al atoms.

Similar outcomes were observed when the initial H^+^ species targeted the O7 position, while the second H^+^ interacted with different sites (Table S4). The most favorable configuration involves the second proton attacking the O8 position (RMS = 0.715 Å and second H+ PRE = −959 kJ/mol) (Figure 9), accompanied by a significant distortion: the Al(V)–O8 and Al(II)–Al(V) bonds lengthen, resulting in the largest PRE for this series (−1924 kJ/mol).

Suppose the first proton attacks O5 (Table S4). In that case, the resulting configurations of the second attack, regardless of the target oxygen, are the least distorted (overall RMS = 0.374 Å) and have the lowest 2H^+^ PRE values (average −1758 kJ/mol). Consequently, this combination is probably the least efficient for the protolysis reaction.

If the initial proton attack comes from an H_3_O^+^ (Table 5 and Table S5), the resulting configurations from the second H^+^ attack resembled those previously described for two protons. However, the average PRE values for the H_3_O^+^ plus H^+^ attack were lower than those for the earlier two-proton attack. In fact, when comparing H_3_O^+^–H^+^ attack to H^+^–H^+^ attack to this face, the PREs for the second proton attack are similar. Processes involving H_3_O^+^ ions produce the lowest PRE values, but the overall face RMS of the geometric distortions remains comparable in magnitude (0.745 Å and 0.678 Å, respectively).

### 2.3. {110} Edge Surface

**Figure 10:**
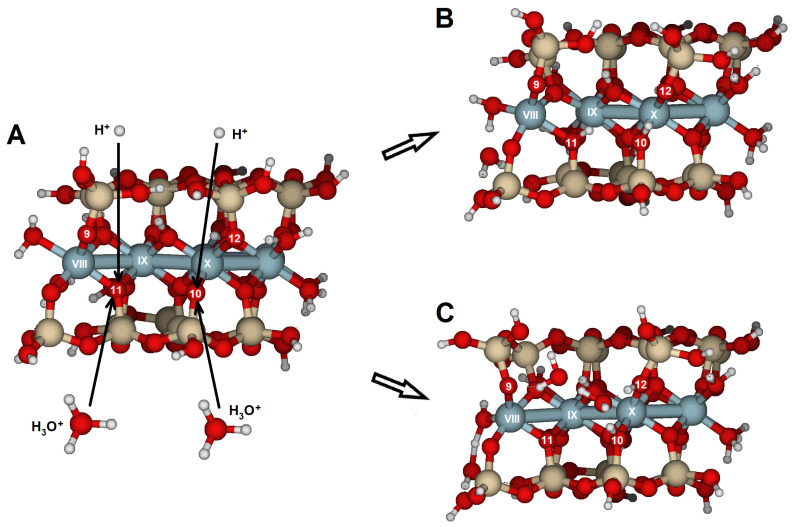
The most stable optimized structures of edge facets in pyrophyllite parallel to the {110} lattice planes: (**A**) before proton attack; (**B**) after attack with two protons across O10 and O11; and (**C**) when protonation occurs with H_3_O^+^ across O10 and O11. Numbers label potential reactive O atoms, and Roman numerals identify Al atoms.

Reactions on the surface parallel to the {110} face were achieved through oxygens O9, O10, and O11 (Figure 3A,B). O9 and O10 are bridge aluminol oxygens (Si–O–Al), and O11 is a bridge oxygen (Si–O < Al_2_). The results of oxygen protonation are listed in Table 6 and Table S6, and Figure 10. No significant distortions were observed, corresponding with an overall RMS of 0.087 Å, which is much lower than the overall RMS values obtained for reactions on the {100} and {010} faces. Protonation at O11, a bridge oxygen, should show a higher RMS value; however, since O11 is located on this face, no substantial distortion was observed. They indicate the greater basicity of these oxygens, although their protonation does not cause any bond-breaking reactions on this face.

Calculations substituting H^+^ with H_3_O^+^ yielded similar results but slightly lower RMS values compared to H^+^ attack (Table 6). In the case of hydronium attack, the Al(X)–O10 distance increased by about 0.14 Å, regardless of which oxygen held the proton. O10 is the outer oxygen on the edge. The attack on O9 or O10 results in a similar configuration.

In the case of a second protonation, either H^+^ or H_3_O^+^ caused elongations in the bonds near O10 and O11, but did not result in significant structural distortion or bond breakage (Table 7). For the H^+^ attack, the greatest elongations were observed in the bond Al(IX)–O11, as well as in the distance between Al(VIII) and Al(IX). This oxygen is a bridge oxygen; therefore, protonation occurred at the lone electron pair, making this oxygen unstable due to the excess positive charge. However, the structural distortion resulting from protonation is insufficient to trigger the protolysis reaction.

The structural distortions were even less pronounced when the attack involved two hydronium ions (Table 7 and Table S7, Figure 10). Structural changes were limited (overall RMS = 0.101 Å), with atomic distances similar to those prior to protonations. Additionally, a proton moved from O11 to O12. The bridge aluminol O12, with two lone electron pairs, may better accommodate the proton than the bridge oxygen O11, resulting in the most stable protonated form. Furthermore, water molecules form hydrogen bonds and facilitate the migration of protons between oxygen atoms.

### 2.4. {130} Edge Surface

**Figure 11:**
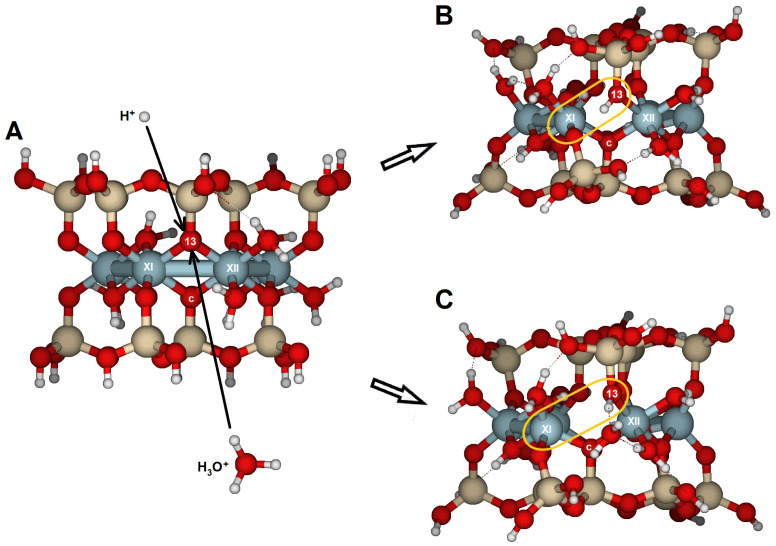
Stable configuration of edge facets in pyrophyllite aligned with the {130} lattice planes: (**A**) before protonation, (**B**) after attack with a H^+^ at O13; and (**C**) following attack by H_3_O^+^ at O13. The yellow ovals indicate the elongated bond after attack: Al(XI)-O13. Numbers label potential reactive O atoms, and Roman numerals identify Al atoms.

On the {130} face, only the bridge oxygen O13 was susceptible to reaction (Figure 3C,D). The structure optimization for a proton attack showed geometrical transformations with an RMS of 0.356 Å and elongation of the Al(XI)–O13 bond from 2.010 to 2.707 Å (Table 8, Figure 11). The PRE for this oxygen is −930 kJ/mol, similar to those derived for the {100} face. The increase in the Al(XII)–O13 bond from 1.940 to 2.198 Å and the Al(XI)···Al(XII) distance from 3.008 to 3.294 Å were much less significant. Protonation of O13 clearly triggered protolysis and dissociation of the structure, with Al(XI) in fivefold coordination. The attack of O13 by hydronium enhanced the transformations compared with the protons. The RMS reached 0.650 Å, and the Al(XI)–O13 distance enlarged to 3.353 Å. The water molecule remained near Al(XII) and formed a hydrogen bridge with the protonated O13. The fivefold coordination of the Al(XI) atom made it less stable. The water molecule enables a more open structure compared to a single proton attack. Protonation of this edge led to the structure disruption.

## 3. Discussion

A systematic study of the initial steps of the dissolution reaction was performed by modeling the protolysis of the {100}, {010}, {110}, and {130} surfaces in molecular cluster models of pyrophyllite. These surfaces are attacked by either H^+^ or H_3_O^+^, which can be considered the first steps of hydrolysis in acidic media. Before reaction, the dangling atoms produced by cutting the structure were relaxed with H or H_2_O. The reactive oxygens corresponded to those in the octahedral sheet, either as bridge oxygens (Si–O < Al_2_), bridge aluminol oxygens (Si–O–Al), or hydroxyl groups (Al–OH–Al).

We should remember that the molecular clusters are calculated in a vacuum. Surface structure calculations were done without an explicit bulk water phase, which could influence the relative stability of surface proton configurations. Under natural conditions, dangling atoms at the surface interface relax through interactions with solvent molecules that may be adsorbed at specific sites. Oxygen atoms on the surface can carry a local excess of negative charge, although the entire surface remains neutral in non-protonated or non-hydronium structures. Martins et al. [32] reported strong interactions between water molecules and edge surfaces, with some water molecules being trapped at particular Al sites—especially those with low coordination—which aligns with our observations in the molecular models.

Furthermore, bridge aluminol oxygens act as Brønsted bases due to two lone electron pairs and an excess negative charge (Si–O^δ-^–Al). These correspond to bridge oxygens on the outermost face where one Al atom is missing; in aqueous media, relaxation of these sites may lead to protonation of the oxygens, which have only one lone electron pair and carry an excess positive charge (Si–O^δ+^H–Al). Such reactive groups function as amphoteric surface sites in water [17,36,37].

The most reactive oxygens were the bridge oxygens, whose coordination sphere is completed with one Si and two Al, especially those located at the inner part of the edge. These oxygens have only a lone electron pair that a proton can attack. That is the case with O2 and O4 on the {100} face, or O5 on the {010} face. This protonation renders the oxygen atom unstable, causing bond breaking (Figure 4 and Figure 8). The results from modeling the four selected faces showed them as the most reactive oxygens vulnerable to proton attack, as well as highlighting the reactivity differences among the faces.

The third type of oxygen, the hydroxyl groups (Al–OH–Al), such as O*a*, O_b_, and O*c*, do not protonate in our model calculations. Hydroxyl groups have a passive role in protolysis. After protonation of a bridge oxygen (e.g., O2, Figure 4B), the hydroxyl groups help accommodate the structural distortions by reorganizing the Al–O bonds. This reorganization facilitates the dissociation of the tetrahedral and octahedral sheets, allowing the structure to open and leading to the breaking/hydrolysis of the structure.

The optimized configurations resulting from protonation reactions on the four studied edge faces led to protolysis at three of the edges: {100}, {010}, and {130}. However, the {110} face accommodated protons with minimal structural distortions, and no bonds were broken. The average values of the PRE (∆E_i_) and the overall structural distortion (RMS calculated for all configurations in a series) for each face after one or two protons were added are highlighted. These results emphasize the unique behavior of the {110} face. The oxygens on this edge exhibited the highest PRE values of all the faces, but some of the lowest overall RMS of structural changes. These findings identify the {110} face as the least reactive among the four edges, which, according to Wulff’s rule, should be the predominant face in equilibrium crystals. This conclusion aligns with the morphology of idiomorphic pyrophyllite crystals (e.g., [31]).

The {110} edge consists of a chain of octahedra (Al) sandwiched between two chains of tetrahedra (Si), arranged in a zig-zag pattern (Figure 1A). The three types of oxygen are exposed (Figure 3A,B). The optimized cluster configurations show their high PRE and the edge’s capacity to protonate with minimal structural distortions (Table 6 and Table 7; Figure 10). The water molecules released after the dissociation of hydronium ions and the adsorption of protons appear to connect to the edge by completing the Al coordination sphere, while also enabling protons to migrate between oxygens through hydrogen bonds. These findings highlight the active role of water molecules in silicate dissolution by adsorption and as carriers of proton mobility. Likely, breaking bonds would have required prior protonation reactions, increased the positive charge density, and caused more severe structural distortions. Nonetheless, in natural conditions, with a complete set of water molecules in the hydration sphere, spare water molecules can also enter the solvation sphere and have no impact on the system stability.

Protonation reactions at the {010} face caused significant structural distortions and protolysis (Figure 8). The proton attack is most effective at O5, while the hydronium attack is more effective at O8. In both cases, protonation leads to broken bonds, with similar RMS values (Table 3). However, an attack with hydronium results in Al(V) with 4-fold coordination. The optimized structure is significantly more open than after a single proton attack, with water molecules forming multiple hydrogen bonds. A configuration similar to that obtained after one hydronium attack required two protons, as shown in Figure 9B and Table 3 and Table 4, highlighting the key role of water molecules during hydrolysis.

The {010} and {110} edges are quite similar (Figure 1A) in terms of atom distribution. In {110} faces, two hydroxyl groups are exposed at the solid/solution interface. They bond with Al(IX) and Al(X) (Figure 3A,B). However, hydroxyl groups are not exposed on the {010} faces (Figure 2C,D). As mentioned earlier, hydroxyl groups are not reactive but help accommodate structural distortions caused by protonation reactions. This slight difference may explain the varying reactivity of the two edges. Although the {110} surface has the highest PRE among all the crystal edges, it can still accommodate structural distortions without protolysis. Therefore, the {110} face is *de facto* the least reactive edge.

The {100} face appears to be more reactive than the {010} face, with slightly higher average PRE and lower overall RMS values for one- and two-proton attacks. The RMS values indicate that structural distortions are significant but more limited than at {010}. The zig-zag arrangement of Al atoms makes the edge relatively rigid. However, the {100} edge is formed by a succession of hemihexagonal octahedral cavities (Figure 1A), which makes the edge more flexible and capable of accommodating distortions. The {100} face contains four bridge oxygens (e.g., O2 and O4) and two hydroxyls (O*a*, O*b*) within the hexagonal cavity, along with four outer bridge aluminol oxygens (e.g., O1, O3). Protonation of O1 and O3 alone is not enough to trigger hydrolysis. Protolysis becomes effective after protonation of bridge oxygens (Si–O < Al_2_), although all protonations contribute to some extent. It is difficult to explain why O2 has a higher PRE than O4 (Table 1). The optimized Al–O distances before protonation are slightly longer for O2 than for O4. Attack at O2 results in greater distortion, involving the breaking of the Al(IV)–O2 bond and the rearrangement of additional bonds, including the hydroxyl O*b*. In contrast, the attack at O4 only results in the breaking of the Al(III)–O4 bond and a slightly less distorted structure. The second protonation increases distortion, leading to a more open hemihexagonal octahedral cavity. The most favorable combinations include a bridge oxygen (O2, O4) and a bridge aluminol oxygen (O1, O3). Clearly, the attack by a proton (or hydronium ion) triggers the hydrolysis of pyrophyllite. Additionally, bond breaking helps to relax the structural stress, resulting in a lower RMS value for the {100} edge compared to the {010} edge. Therefore, the {100} edge is a more reactive face than the {010}.

The PRE of the reactive oxygen (O13) at the {130} face is the lowest of the average values (Table 8). The reaction is efficient, breaking the Al(XI)–O13 bond and opening the area attacked. These values and the optimized cluster configurations indicate that the {130} face is more reactive than the {110} face.

Only two reactive oxygens were identified in the cluster used to model the {130} edge. However, this might be an artifact of the small cluster size, which was limited to keep computational requirements manageable. Figure 1A shows a similar arrangement of Al atoms on the {130} and {100} faces, featuring a series of hemihexagonal octahedral cavities. Differences result from the relative positions of tetrahedral and octahedral sheets. Although the edges are not identical, the reactive sites on both faces may be similar. Remember that for one-H^+^-attack, the overall RMS of {130} is higher than {100}. Therefore, similar reactivity could be expected for faces {100} and {130}. Additionally, the modeling results for both faces may complement each other. The Al(XI) and Al(XII) might correspond to Al(IV) and Al(I), respectively, in clusters modeling the {130} and {100} edges. The Al(II) and Al(III) in {100} could provide insights into the reactivity of oxygens attached to the Al atoms in the innermost hemihexagonal cavity of the {130} face. Based on these points, the reactivity of the {130} edge may resemble that of the {100} edge.

The relative reactivities of the studied faces are approximately {100} ~ {130} > {010} >> {110}. This sequence aligns with Bleam et al.’s [16] calculation, which indicated that forming {100} and {130} edges required about 16% more energy compared to {010} and {110}. According to Wulff’s rule, the particle morphology is primarily determined by the most stable or less reactive faces, which, in the case of pyrophyllite, are {010} and {110} [31].

Modeling the attack with a hydronium ion instead of a proton results in the protonation of a reactive oxygen and the release of a water molecule. This water molecule stays nearby and helps relax the local excess or deficit of charge. Protonation caused by one H^+^ attack leads to notable structural distortions. In contrast, the H_3_O^+^ attack is usually more effective because water forms hydrogen bonds, completing the coordination sphere of hydrolyzed aluminum atoms, or acting as a pathway for proton migration between nearby oxygens. The PREs for hydronium attacks are about one-third of those for H^+^, and the distortion caused is similar, as shown by comparable overall RMS values. The positioning of water molecules near the affected area likely helps stabilize the hydration sphere, bringing it back to the normal surface hydration level. These findings highlight the critical role of water molecules in dissolution reactions.

Generally, the second protonation results in higher RMS values than the first. The attack of two protons (or a hydronium and a proton), whether simultaneous or sequential, begins on a highly reactive bridge oxygen. This increases the reactivity of other oxygens, such as the outer bridge aluminol oxygens, and promotes structural breakdown by splitting the octahedral and tetrahedral sheets. The protolysis mainly affects the octahedral cations, which are gradually surrounded by water molecules before being released into the solution. Fully releasing aluminum into solutions would require additional calculations, including accounting either for water molecules or a continuum solvent method to model the hydration layer surrounding the crystal. These second protonations, which have the highest overall RMS values, suggest that reducing the pH and increasing the temperature would promote pyrophyllite dissolution; highly acidic conditions might cause multiple proton attacks around reactive sites.

Although transition states between reactants and products have been studied, they have not yet been observed. This indicates that protolysis occurs directly, without an intermediate step. Consequently, no energy barrier was detected in any of the protolysis reactions, which is consistent with the molecular dynamics results of Schilemann and Churakov [34]. Surface protonation reactions are reversible at amphoteric surface sites. Surface protonation shows very little temperature dependence [37]. They suggested that the slight variations in proton adsorption constants likely stem from changes in the water dissociation constant (K_w_) with temperature. This behavior aligns with a very low energy barrier as suggested by DFT calculations.

## 4. Materials and Methods

### 4.1. Surface Protonation Reactions

Efforts have been made to model the interaction of the proton/hydronium with different atoms on the modeled faces, aiming to identify which surface is more reactive during the initial stages of dissolution. Therefore, the PRE of these surfaces can be used to estimate the protolysis reaction through an energy balance. The following reaction will model a proton attack:

S + H^+^ → S − H^+^ + ∆E_1_, (1)

being S_,_ the surface, and ∆E_i_ is the total energy balance between products and reactants for reaction *i*, that is, the PRE. For the reaction with a hydronium:

S + H_3_O^+^ → H_2_O − S − H^+^ + ∆E_2_, (2)

where the H_2_O molecule of the H_3_O^+^ also remains linked to the molecular cluster model.

Furthermore, a second set of PREs can also be modeled for attacking with a second proton on the protonated surface in Equation (1):

S − H^+^ + H^+^ → S − H_2_^2+^ + ∆E_3_ (3)

or adding to the hydrated surface a second proton ion in Equation (2):

H_2_O − S − H^+^ + H^+^ → H_2_O − S − H_2_^2+^ + ∆E_4_. (4)

Although reactions (3) and (4) are viewed as part of the second step in a step-by-step process, an attempt has also been made to consider a single-step reaction involving two protons by summing the reaction energies of the individual reactions, such as:

S + 2H^+^ → S − H_2_^2+^ + ∆E_5_, (5)

S + H_3_O^+^ + H^+^ → H_2_O − S − H_2_^+^ + ∆E_6_. (6)

It is well known that protons exist as hydronium or polyhydrated ions in water [38]. Surface reactions were calculated in a vacuum without an explicit bulk water phase. A single proton was considered a simpler species, and its behavior was compared with that of hydronium.

### 4.2. Structural Models

Different molecular cluster models of pyrophyllite faces were proposed to investigate the protolysis mechanism of dioctahedral phyllosilicates. Pyrophyllite is an ideal prototype for the 2:1 dioctahedral phyllosilicates because the absence of any charge coming from isomorphic substitutions reduces the complexity (Figure 1A). The pyrophyllite basal planes could be considered inert [29]. Different molecular cluster models have been considered as the four most representative edge surfaces of mineral structure. The pyrophyllite has the ideal formula (Al_2_□)(Si_4_)O_10_(OH)_2_ (where □ in the formula means one octahedral vacant cavity). The models were based on two hexagonal cavities (rings of SiO_4_ units) of tetrahedral sheets, which were joined by one octahedral sheet. It can be assumed that the most stable edge faces are related to cleavage planes, which minimize the number of dangling bonds and leave the relevant atoms close to the edge face, in accordance with basic crystallographic principles. Furthermore, these representative faces are characterized by the maximum atom density [15,16,30,31]. Adapting these principles, the molecular cluster models have been designed by cutting the structure through different planes: (100), (010), (110), and (130) (Figure 1B).

- Si_16_Al_6_O_56_H_30_ · 2H_2_O was proposed to study the {100} and {010} surfaces (Figure 2);
- Si_17_Al_7_O_62_H_35_ · H_2_O for the {110} surface (Figure 3A,B); and
- Si_14_Al_6_O_56_H_30_ · 2H_2_O for the {130} surface (Figure 3C,D).

Protons and water molecules were added to the models at the dangling atoms, when necessary, to close the dangling bonds and maintain the coordination of the octahedral cations. These additions were made because larger clusters would be computationally very demanding, and finally dangling bonds have to be closed. Additionally, protolysis effects can be locally highlighted with sufficient detail to be relevant in these relatively small cluster models. The optimized structure of the edge-sharing dioctahedral cluster is shown in Figure 1B, where dashed lines mark the main edge faces studied.

In these surface models, the potentially reactive atoms to be attacked by protons or hydronium ions are either bridge oxygens or oxygens of the hydroxyl groups:

1. {100}: On this configuration, the accessible and non-equivalent oxygen atoms occupied positions O1, O2, O3, and O4 (Figure 2A,B). Two types of oxygens can be found on this surface: (1) oxygens O1 and O3 are bridge aluminols (Si-O-Al-type group), bonded to Al(I) and one Si, and closed either with a H or a H_2_O; and (2), oxygens O2 y O4 correspond to bridge oxygens, bonded to one silicon and two aluminum atoms [Al(III) and Al(IV)] (Si-O < Al_2_), located inside the octahedral cavity.
2. {010}: On this edge surface, the reactive atoms are four bridge oxygens (O5, O6, O7, and O8, all of which are Si-O < Al_2_ type). (Figure 2C,D).
3. {110}: This face contains three positions. O9 and O10 are bridge aluminol oxygens (Si-O-Al), and O11 is a bridge oxygen (Si-O < Al_2_ type, Figure 3A,B).
4. {130}: On this face, the possibilities are reduced to O13 (Si-O < Al_2_), as shown in Figure 3C,D.

### 4.3. Computational Methods

Calculations were carried out using Gaussian 16 software [39]. CP of the PES of the protolysis reaction were calculated at the Becke three-parameter hybrid correlation exchange functional (B3LYP) [40]. This functional shows good performance on many electron and molecular properties [19,41,42,43], Considering the number of atoms and the nature of the atoms in the clusters, the 3-ξ basis set with diffuse and polarization functions would be very computationally demanding, and for this reason it is suitable to use effective core potentials, included in the LANL2DZ basis set (Los Alamos National Laboratory2 double-ξ) [44,45,46,47], which is a good compromise between quality and calculation time. To determine the CP (i.e., equilibrium geometries and transition structures) of the PES, the Berny method [48] was employed for the optimization procedure, and a full optimization process was performed for all calculated models. Thresholds in root mean square of maximum forces and displacements of internal coordinates were 0.00045 and 0.0003 Hartree/Bohr, and 0.0018 and 0.0012 Bohr, respectively. Our CP showed values below these limits. Once the reactant and products were optimized and a CP was obtained, from their energies, the PREs were obtained by difference, following Equations (1)–(6).

## 5. Conclusions

DFT modeling of the reactivity of the four main pyrophyllite edge faces under acidic conditions, represented by proton and/or hydronium attack, allowed the identification of face {110} as the most stable. The reactive oxygens correspond to the bridge aluminol oxygens (Si–O–Al). These oxygens can be protonated and accommodate the positive charge and structural distortions without causing protolysis of nearby bonds. Effective protolysis requires the attack of additional protons/hydroniums. This situation corresponds to more acidic conditions, that is, a lower pH.

In contrast, the other three faces, {100}, {130}, and {010}, exhibited higher reactivity. DFT calculations showed that this increased reactivity was associated with the presence of bridge oxygen groups (Si–O < Al_2_) linked to Al cations located in the second line. Once oxygen is protonated, the structure destabilizes and evolves by breaking a bond, initiating the separation of the octahedral and tetrahedral sheets, and ultimately leading to the hydrolysis and detachment of the octahedral cations. In addition, it was demonstrated that hydroxyl groups (Al–OH–Al) have a low protonation capacity and are therefore less reactive.

To conclude, we should focus on the primary goal of this study, which is to gain a deeper understanding of the protolysis reaction involved in the dissolution mechanisms of dioctahedral phyllosilicates under acidic conditions and to gather atomistic evidence of the initial steps of these processes. Pyrophyllite is proposed as a simplified model for dioctahedral phyllosilicates. Experimental methods cannot identify individual steps in the dissolution process. Dissolution experiments showed that the most reactive surfaces are the crystal edges, where proton adsorption triggers the process. DFT results are consistent with clay mineral titration experiments that concluded that reactive groups are located on bridge oxygens (Si–O < Al_2_), which are much more reactive than bridge aluminol (Si–O–Al) and hydroxyl groups (Al–OH–Al) [38,49]. Protonation of these bridge oxygens initiates hydrolysis, confirming that hydrolysis of the bridge oxygens is the rate-limiting step (e.g., [6,9]). Protonation of bridge aluminol oxygens on the outermost edge surface induces and enhances the protonation of bridge oxygens, as shown by surface titration experiments [37,38]. When hydronium ions react instead of protons, they produce similar or greater structural changes, and water molecules form hydrogen bonds with the resulting structures. During dissolution experiments of dioctahedral phyllosilicates, octahedral cations are preferentially released [6,9,50,51,52,53]. This behavior results from the preferential hydrolysis of the bond linking octahedral cations to the tetrahedral sheets, as demonstrated in the modeling reported here.

Finally, we can see once again that the basic surface properties of the local area can be studied with molecular surface models, providing new insights at the atomistic level on the reactivity of these surfaces. Future research should model the dissolution using extended or periodic surface models, which better approximate the actual mineral/solution interface, including the role of the solvent (water). It will also be essential to explore the contribution of isomorphic substitutions present in most phyllosilicates. The atomistic evidence, primarily obtained from molecular models, will be beneficial in further understanding the role of phyllosilicates in various geological and environmental processes.

## Figures and Tables

**Table 1:** RMS (Å) and energy balance of the protonation, PRE (kJ/mol), between the initial and protonated structures, calculated for the {100} edge face with H^+^ and H_3_O^+^ placed on O1, O2, O3, and O4 (Figure 2A). PRE is expressed here as ∆E_i_ according to Equations (1)–(6). The atomic distances of the optimized initial and attacked molecular clusters are listed in Table S1.

{100} Edge Face	H^+^				H_3_O^+^			
	O1	O2	O3	O4	O1	O2	O3	O4
RMS (Å)	0.066	0.420	0.052	0.265	0.048	0.424	0.061	0.261
Overall RMS (Å)	0.252				0.252			
* ∆E_1_, ∆E_2_	−978	−1030	−994	−971	−343	−375	−323	−410
Average ∆E_1_, ∆E_2_	−993				−363			

* Subindexes at ∆E correspond to the equations in Section 4.1.

**Table 2:** RMS (Å) and energy balance of protonation, PRE (kJ/mol), between the initial and protonated structures, calculated for the {100} edge face, with the first H^+^ and H_3_O^+^ placed on O1, O2, O3, and O4, and various options for a second H^+^ attack (Figure 2A). PRE is expressed as ∆E_i_ according to Equations (1)–(6). The atomic distances of the optimized initial and attacked molecular clusters are listed in Table S2.

**{100} Edge Face**	**1st H^+^ on O1**			**1st H^+^ on O2**		
2nd H^+^ on	O2	O3	O4	O1	O3	O4
RMS (Å)	0.829	0.633	0.310	0.084	0.053	0.440
Overall RMS (Å)		0.628			0.260	
* ∆E_3_ (1H^+^)	−983	−851	−771	−774	−741	−782
Average ∆E_3_ (1H^+^)		−868			−766	
∆E_5_ (2H^+^)	−1960	−1829	−1749	−1804	−1771	−1812
Average ∆E_5_ (2H^+^)		−1846			−1796	
	**1st H^+^ on O3**			**1st H^+^ on O4**		
2nd H^+^ on	O1	O2	O4	O1	O2	O3
RMS (Å)	0.122	0.632	0.382	0.969	0.498	0.172
Overall RMS (Å)		0.432			0.637	
* ∆E_3_ (1H^+^)	−702	−863	−769	−1004	−907	−785
Average ∆E_3_ (1H^+^)		−778			−898	
∆E_5_ (2H^+^)	−1697	−1857	−1763	−1975	−1878	−1756
Average ∆E_5_ (2H^+^)		−1772			−1870	

* Subindexes at ∆E correspond to the equations in Section 4.1.

**Table 3:** RMS (Å) and energy balance of protonation, PRE (kJ/mol), between the initial and protonated structures, calculated for the {010} edge face, by bonding H^+^ and H_3_O^+^ on O5, O6, O7, and O8 (Figure 2C). PRE is expressed as ∆E_i_ according to Equations (1)–(6). The atomic distances of the optimized initial and attacked molecular clusters are listed in Table S3.

{010} Edge Face	H^+^				H_3_O^+^			
	O5	O6	O7	O8	O5	O6	O7	O8
RMS (Å)	0.961	0.248	0.541	0.434	0.571	0.188	0.474	0.948
Overall RMS (Å)	0.605				0.609			
* ∆E_1_, ∆E_2_	−1024	−1010	−965	−902	−366	−275	−394	−261
Average ∆E_1_, ∆E_2_	−975				−324			

* Subindexes at ∆E correspond to the equations in Section 4.1.

**Table 4:** RMS (Å) and energy balance of protonation, PRE (kJ/mol), between the initial and protonated structures, calculated for the {010} edge face, with the first H^+^ on O5, O6, O7, and O8, and various possibilities for the second attached H^+^ (Figure 2C). PRE is expressed as ∆E_i_ according to Equations (1)–(6). The atomic distances of the optimized initial and attacked molecular clusters are listed in Table S4.

**{010} Edge Face**	**1st H^+^ on O5**			**1st H^+^ on O6**		
2nd H^+^ on	O6	O7	O8	O5	O7	O8
RMS (Å)	0.204	0.502	0.355	1.025	1.260	0.591
Overall RMS (Å)	0.374			0.998		
* ∆E_3_ (1H^+^)	−712	−788	−702	−852	−719	−852
Average ∆E_3_ (1H^+^)		−734			−807	
∆E_5_ (2H^+^)	−1736	−1812	−1727	−1861	−1728	−1861
Average ∆E_5_ (2H^+^)		−1758			−1817	
	**1st H^+^ on O7**			**1st H^+^ on O8**		
2nd H^+^ on	O5	O6	O8	O5	O6	O7
RMS (Å)	0.389	0.272	0.715	0.614	1.162	0.886
Overall RMS (Å)	0.495				0.915	
* ∆E_3_ (1H^+^)	−802	−756	−959	−1056	−895	−778
Average ∆E_3_ (1H^+^)		−839			−910	
∆E_5_ (2H^+^)	−1768	−1721	−1924	−1958	−1796	−1680
Average ∆E_5_ (2H^+^)		−1804			−1811	

* Subindexes at ∆E correspond to the equations in Section 4.1.

**Table 5:** RMS (Å) and energy balance of protonation, PRE (kJ/mol), between the initial and protonated structures, calculated for the {010} edge face, with H_3_O^+^ placed on O5, O6, O7, and O8, and various possibilities for the second attached H^+^ (Figure 2C). PRE is expressed as ∆E_i_ according to Equations (1)–(6). The atomic distances of the optimized initial and attacked molecular clusters are listed in Table S5.

**{010} Edge Face**	**H_3_O^+^ on O5**			**H_3_O^+^ on O6**		
2nd H^+^ on	O6	O7	O8	O5	O7	O8
RMS (Å)	0.747	0.155	0.813	1.423	0.472	0.967
Overall RMS (Å)	0.644			1.030		
* ∆E_4_ (1H^+^)	−766	−764	−667	−821	−807	−937
Average ∆E_4_ (1H^+^)		−733			−855	
∆E_6_ (H_3_O^+^+1H^+^)	−1132	−1130	−1033	−1096	−1082	−1213
Average ∆E_6_ (H_3_O^+^+1H^+^)		−1098			−1130	
	**H_3_O^+^ on O7**			**H_3_O^+^ on O8**		
2nd H^+^ on	O5	O6	O8	O5	O6	O7
RMS (Å)	0.480	0.095	0.765	0.460	0.161	0.156
Overall RMS (Å)	0.524			0.295		
* ∆E_4_ (1H^+^)	−786	−780	−686	−781	−856	−922
Average ∆E_4_ (1H^+^)		−751			−853	
∆E_6_ (H_3_O^+^+1H^+^)	−1180	−1175	−1080	−1042	−1117	−1184
Average ∆E_6_ (H_3_O^+^+1H^+^)		−1145			−1114	

* Subindexes at ∆E correspond to the equations in Section 4.1.

**Table 6:** RMS (Å) and energy balance of protonation, PRE (kJ/mol), between the initial and protonated structures, calculated for the {110} (Figure 3A) edge face, with H^+^ and H_3_O^+^ placed on O9, O10, and O11. PRE is expressed as ∆E_i_ according to Equations (1)–(6). The atomic distances of the optimized initial and attacked molecular clusters are listed in Table S6.

{110} Edge Face	H^+^			H_3_O^+^		
	O9	O10	O11	O9	O10	O11
RMS (Å)	0.088	0.089	0.086	0.061	0.061	0.064
Overall RMS (Å)	0.087			0.062		
* ∆E_1_, ∆E_2_	−1102	−1167	−1134	−479	−479	−493
Average ∆E_1_, ∆E_2_		−1134			−484	

* Subindexes at ∆E correspond to the equations in Section 4.1.

**Table 7:** RMS (Å) and energy balance of protonation, PRE (kJ/mol), between the initial and protonated structures, calculated for the {110} (Figure 3A) edge faces by placing two H^+^ or two H_3_O^+^ on O10 and O11. PRE is expressed as ∆E_i_ according to Equations (1)–(6). The atomic distances of the optimized initial and attacked molecular clusters are listed in Table S7.

{110} Edge Face	2 H^+^	2 H_3_O^+^
RMS (Å)	0.235	0.101
∆E	−1904	−726

**Table 8:** RMS (Å) and energy balance of protonation, PRE (kJ/mol), between the initial and protonated structures, calculated for the {130} (Figure 3C) edge face by placing one H^+^ or one H_3_O^+^ on O13. PRE is expressed as ∆E_1_ according to Equation (1). The atomic distances of the optimized initial and attacked molecular clusters are listed in Table S8.

{130} Edge Face	H^+^	H_3_O^+^
	O13	O13
RMS (Å)	0.356	0.650
∆E_1_	−930	−297

Subindexes at ∆E correspond to the equations in Section 4.1.

## Data Availability

The original contributions presented in this study are included in the article. For further inquiries, please contact the corresponding authors.

## Supplementary Materials

The following supporting information can be downloaded at: https://www.mdpi.com/article/10.3390/molecules30234530/s1, Tables S1–S8: Atomic distances of the optimized initial and attacked molecular clusters (Å), calculated on the corresponding edge face with H^+^ and/or H_3_O^+^ placed on the indicated oxygens. It includes RMS between the initial and protonated structures, the overall RMS across different protonations for the series, and the energy balance of the protonation (kJ/mol). Bold distances indicate the most changed distances from the initial ones. PRE is written here as ∆Ei according to Equationss (1)–(6).

## Abbreviations

The following abbreviations are used in this manuscript:

B3LYP	Becke three-parameter hybrid correlation exchange functional
CP	Critical points
DFT	Density functional theory
LANL2DZ	Los Alamos National Laboratory2 double-ξ
PBC	Periodic bond chain
PES	Potential energy surface
PRE	Proton reaction energy
RMS	Root mean square
TOT	Tetrahedral–octahedral–tetrahedral sheet arrangement in phyllosilicates
