# Supplementary material for: Protolysis Reaction on Pyrophyllite Surface Molecular Models: A DFT Study
Source: Molecules. 2025 Nov 24;30(23):4530. doi: 10.3390/molecules30234530 (PMC12692931; doi:10.3390/molecules30234530)
Supplement: Supplementary file 1 [file molecules-30-04530-s001.zip › molecules-3959989-supplementary.docx]

Protolysis reaction on pyrophyllite surface molecular models: a DFT study

SUPPLEMENTARY MATERIAL

María Bentabol ª, Carlos Pérez del Valle ^b^, Alfonso Hernández-Laguna ^c^, F. Javier Huertas ^c^

^a^ Departament of Inorganic Chemistry, Crystallography and Mineralogy. Facultad de Ciencias. Universidad de Málaga. Campus de Teatinos. 29071 Málaga (Spain)

^b^ Department of Molecular Chemistry, Université Grenoble Alpes, F-38058, (Grenoble), France

^c^ Instituto Andaluz de Ciencias de la Tierra (IACT-CSIC), Consejo Superior de Investigaciones Científicas. 18100 Armilla (Granada), Spain

Authors emails:

bentabol@uma.es; [carlos.Perez@univ-grenoble-alpes.fr](mailto:carlos.Perez@univ-grenoble-alpes.fr); [a.h.laguna@csic.es](mailto:a.h.laguna@csic.es); javier.huertas@csic.es

Correspondence to: Alfonso Hernández-Laguna (a.h.laguna@csic.es), María Bentabol (bentabol@uma.es)

Table S1. Atomic distances of the optimized initial and attacked molecular clusters (Å), calculated on the {100} edge face with H^+^ and H_3_O^+^ placed on O1, O2, O3, and O4 (Figure 2A). It includes RMS between the initial and protonated structures, the overall RMS across different protonations for the series, and the energy balance of the protonation (kJ/mol). Bold distances indicate the most changed distances from the initial ones. PRE is written here as ∆E_i_ according to Eqs. (1) – (6).

| **Atomic distances** |  | **H^+^** | | | |  | **H_3_O^+^** | | | |
| --- | --- | --- | --- | --- | --- | --- | --- | --- | --- | --- |
|  | Initial | Optimized | | | |  | Optimized | | | |
|  |  | O1 | O2 | O3 | O4 |  | O1 | O2 | O3 | O4 |
| Al(I)-O1 | 1.850 | 2.007 | 1.819 | 1.866 | 1.845 |  | 1.971 | 2.007 | 1.866 | 1.912 |
| Al(I)-O3 | 1.853 | 1.840 | 1.861 | 1.983 | 1.856 |  | 1.922 | 1.971 | 1.912 | 1.889 |
| Al(I)-O5 | 1.906 | 1.809 | 1.958 | 1.859 | 1.935 |  | 1.877 | 1.896 | 1.895 | 1.937 |
| Al(I)-O6 | 1.888 | 1.846 | 1.892 | 1.827 | 1.890 |  | 1.860 | 1.982 | 1.881 | 1.945 |
| Al(II)-O5 | 2.004 | 2.168 | 1.904 | 2.026 | 1.938 |  | 2.048 | 1.896 | 2.021 | 1.955 |
| Al(II)-O6 | 2.026 | 2.014 | 1.908 | 2.096 | 2.121 |  | 2.035 | 1.928 | 2.033 | 2.055 |
| Al(II)-O*a* | 1.942 | 1.927 | 1.859 | 1.912 | 2.020 |  | 1.962 | 1.862 | 1.953 | 2.071 |
| Al(II)-O*b* | 1.957 | 1.930 | **2.874** | 1.978 | 1.933 |  | 1.930 | **2.983** | 1.980 | 1.912 |
| Al(III)-O*a* | 1.955 | 1.970 | 1.907 | 1.959 | 1.854 |  | 1.949 | 1.874 | 1.927 | 1.856 |
| Al(III)-O*b* | 1.956 | 1.988 | 1.973 | 1.992 | 1.875 |  | 1.980 | 1.970 | 1.981 | 1.859 |
| Al(III)-O2 | 1.971 | 1.971 | **2.075** | 1.987 | 1.923 |  | 1.979 | **2.095** | **2.005** | 1.922 |
| Al(III)-O4 | 1.935 | 1.928 | 1.983 | 1.933 | **2.952** |  | 1.966 | 1.950 | 1.948 | **2.964** |
| Al(IV)-O2 | 1.986 | 1.983 | **3.337** | 1.982 | 2.121 |  | 1.979 | **3.279** | 1.941 | 2.054 |
| Al(IV)-O4 | 1.972 | 1.993 | 1.935 | 1.977 | 2.053 |  | 1.986 | 1.893 | 1.980 | 2.044 |
| Al(I)-Al(II) | 3.109 | 3.084 | 3.007 | 3.092 | 3.127 |  | 3.081 | 3.052 | 3.012 | 3.118 |
| Al(II)-Al(III) | 3.111 | 3.112 | 3.310 | 3.124 | 3.065 |  | 3.120 | 3.334 | 3.118 | 3.062 |
| Al(III)-Al(IV) | 3.033 | 3.038 | 3.489 | 3.042 | 3.440 |  | 3.041 | 3.427 | 3.031 | 3.329 |
| Al(I)-Al(IV) | 5.766 | 5.875 | 6.206 | 5.638 | 5.780 |  | 5.883 | 6.218 | 5.546 | 5.642 |
| RMS (Å) |  | 0.066 | 0.420 | 0.052 | 0.265 |  | 0.048 | 0.424 | 0.061 | 0.261 |
| Overall RMS (Å) |  | 0.252 | | | |  | 0.252 | | | |
| *∆E_1_, ∆E_2_ |  | -978 | -1030 | -994 | -971 |  | -343 | -375 | -323 | -410 |
| Average ∆E_1_, ∆E_2_ |  | -993 | | | |  | -363 | | | |

* Subindexes at ∆E correspond to the equations in section 4.1.

Table S2. Atomic distances of the optimized initial and attacked molecular clusters (Å) on the {100} edge face, with the first H^+^ placed at O1, O2, O3, and O4, and various options for a second H^+^ at-tack (Figure 2A). RMS between the initial and protonated structures, overall RMS for different protonations for the series, and the energy balance of protonation (kJ/mol) (Equations 3 and 5). Bold distances indicate the most changed distances from the initial ones. PRE are written here as ∆Ei according to Eqs. (1) – (6).

| **Atomic distances** | **1^st^ H^+^ on O1** | | | | **1^st^ H^+^ on O2** | | | |
| --- | --- | --- | --- | --- | --- | --- | --- | --- |
|  | Initial | Optimized | | | Initial | Optimized | | |
| 2nd H^+^ on |  | O2 | O3 | O4 |  | O1 | O3 | O4 |
| Al(I)-O1 | 2.007 | **4.873** | 1.984 | 2.039 | 1.819 | 2.015 | 1.827 | 1.793 |
| Al(I)-O3 | 1.840 | 1.865 | 1.834 | 1.833 | 1.861 | 1.837 | 1.978 | 1.937 |
| Al(I)-O5 | 1.809 | 1.786 | 1.856 | 1.778 | 1.958 | 1.853 | 1.900 | 1.939 |
| Al(I)-O6 | 1.846 | 1.875 | 1.872 | 1.890 | 1.892 | 1.843 | 1.823 | 1.874 |
| Al(II)-O5 | 2.168 | **3.787** | 1.969 | 2.297 | 1.904 | 1.998 | 1.923 | 1.888 |
| Al(II)-O6 | 2.014 | 1.937 | 1.919 | 1.995 | 1.908 | 1.902 | 1.977 | 1.950 |
| Al(II)-Oa | 1.927 | 1.936 | 1.842 | 2.001 | 1.859 | 1.843 | 1.848 | 1.844 |
| Al(II)-Ob | 1.930 | 1.924 | **3.732** | 1.920 | **2.875** | **2.814** | **2.805** | **2.955** |
| Al(III)-Oa | 1.970 | 1.880 | 1.870 | 1.919 | 1.907 | 1.922 | 1.908 | 1.797 |
| Al(III)-Ob | 1.988 | 1.900 | **3.870** | 1.969 | 1.973 | 1.976 | 2.007 | 1.912 |
| Al(III)-O2 | 1.971 | **3.083** | 1.906 | 2.140 | 2.075 | 2.089 | 2.062 | 1.933 |
| Al(III)-O4 | 1.928 | 1.905 | 1.873 | 1.965 | 1.983 | 1.983 | 1.986 | **3.491** |
| Al(IV)-O2 | 1.983 | 2.179 | 2.037 | 1.925 | **3.335** | **3.382** | **3.247** | **3.822** |
| Al(IV)-O4 | 1.993 | 2.101 | 2.068 | **3.124** | 1.935 | 1.934 | 1.940 | 1.888 |
| Al(I)-Al(II) | 3.084 | 2.993 | 2.925 | 3.149 | 3.007 | 2.947 | 2.966 | 3.002 |
| Al(II)-Al(III) | 3.112 | 3.059 | 3.529 | 3.081 | 3.310 | 3.303 | 3.307 | 3.212 |
| Al(III)-Al(IV) | 3.038 | 3.442 | 2.877 | 3.504 | 3.490 | 3.503 | 3.476 | **4.338** |
| Al(I)-Al(IV) | 5.875 | 5.628 | 5.552 | 6.278 | 6.206 | 6.439 | 6.289 | 6.644 |
| RMS (Å) |  | 0.829 | 0.633 | 0.310 |  | 0.084 | 0.053 | 0.440 |
| Overall RMS (Å) |  |  | 0.628 |  |  |  | 0.260 |  |
| *∆E_3_ (1H^+^) |  | -983 | -851 | -771 |  | -774 | -741 | -782 |
| Average ∆E_3_ (1H^+^) |  |  | -868 |  |  |  | -766 |  |
| ∆E_5_ (2H^+^) |  | -1960 | -1829 | -1749 |  | -1804 | -1771 | -1812 |
| Average ∆E_5_ (2H^+^) |  |  | -1846 |  |  |  | -1796 |  |
| **Atomic distances** | **1^st^ H^+^ on O3** | | | | **1^st^ H^+^ on O4** | | | |
|  | Initial | Optimized | | | Initial | Optimized | | |
| 2nd H^+^ on |  | O1 | O2 | O4 |  | O1 | O2 | O3 |
| Al(I)-O1 | 1.866 | 1.971 | 1.876 | 1.895 | 1.845 | **4.830** | 1.807 | 1.813 |
| Al(I)-O3 | 1.983 | 1.959 | 1.968 | 1.972 | 1.856 | 1.855 | 1.874 | 2.121 |
| Al(I)-O5 | 1.859 | 1.812 | 1.839 | 1.850 | 1.935 | 1.741 | 1.960 | 1.918 |
| Al(I)-O6 | 1.827 | 1.810 | 1.868 | 1.857 | 1.890 | 1.877 | 1.889 | 1.742 |
| Al(II)-O5 | 2.026 | 1.998 | 2.097 | 2.026 | 1.938 | **3.743** | 1.910 | 1.920 |
| Al(II)-O6 | 2.096 | 2.141 | 2.023 | 2.096 | 2.121 | 2.005 | 2.114 | **2.743** |
| Al(II)-Oa | 1.912 | 2.104 | 1.907 | 2.048 | 2.020 | 2.037 | 1.958 | 1.910 |
| Al(II)-Ob | 1.978 | 1.899 | 2.055 | 1.927 | 1.933 | 1.892 | 2.025 | 1.909 |
| Al(III)-Oa | 1.959 | 1.960 | 1.927 | 1.893 | 1.854 | 1.888 | 1.812 | 1.877 |
| Al(III)-Ob | 1.992 | 1.966 | 1.878 | 1.969 | 1.875 | 1.867 | 2.041 | 1.900 |
| Al(III)-O2 | 1.987 | 2.021 | **3.417** | 2.108 | 1.923 | 1.832 | 1.841 | 1.906 |
| Al(III)-O4 | 1.933 | 1.976 | 1.846 | 1.978 | **2.952** | **3.411** | **3.663** | **3.000** |
| Al(IV)-O2 | 1.982 | 1.983 | 1.965 | 1.879 | 2.121 | **3.548** | **3.702** | 2.086 |
| Al(IV)-O4 | 1.977 | 1.952 | **3.442** | **3.167** | 2.053 | 1.980 | 1.899 | 2.107 |
| Al(I)-Al(II) | 3.092 | 3.083 | 3.099 | 3.098 | 3.127 | 3.068 | 3.084 | 3.250 |
| Al(II)-Al(III) | 3.124 | 3.128 | 3.092 | 3.098 | 3.065 | 3.079 | 3.109 | 3.005 |
| Al(III)-Al(IV) | 3.042 | 3.020 | 4.752 | 3.394 | 3.440 | 4.897 | 4.562 | 3.402 |
| Al(I)-Al(IV) | 5.638 | 6.087 | 5.831 | 6.652 | 5.780 | 6.325 | 5.444 | 5.883 |
| RMS (Å) |  | 0.122 | 0.632 | 0.382 |  | 0.969 | 0.498 | 0.172 |
| Overall RMS (Å) |  |  | 0.432 |  |  |  | 0.637 |  |
| *∆E_3_ (1H^+^) |  | -702 | -863 | -769 |  | -1004 | -907 | -785 |
| Average ∆E_3_ (1H^+^) |  |  | -778 |  |  |  | -898 |  |
| ∆E_5_ (2H^+^) |  | -1697 | -1857 | -1763 |  | -1975 | -1878 | -1756 |
| Average ∆E_5_ (2H^+^) |  |  | -1772 |  |  |  | -1870 |  |

* Subindexes at ∆E correspond to the equations in section 4.1.

Table S3. Atomic distances of the optimized initial and attacked molecular clusters (Å), on the {010} edge face, by bonding H^+^ and H_3_O^+^ on O5, O6, O7, and O8 (Figure 2C). RMS between the initial and protonated structures, overall RMS across different protonation states for the series, and the energy balance of protonation (kJ/mol) (Equations 1 and 2). Bold distances indicate the most changed distances from the initial ones. PRE are written here as ∆Ei according to Eqs. (1) – (6).

| **Atomic distances** |  | **H^+^** | | | | **H_3_O^+^** | | | |
| --- | --- | --- | --- | --- | --- | --- | --- | --- | --- |
|  | Initial | Optimized | | | | Optimized | | | |
|  |  | O5 | O6 | O7 | O8 | O5 | O6 | O7 | O8 |
| Al(V)-O7 | 2.020 | **3.717** | 2.026 | 2.060 | 1.909 | **3.274** | 2.023 | 1.993 | 1.808 |
| Al(V)-O8 | 1.954 | 1.903 | 2.056 | 2.045 | **3.181** | 1.937 | 2.033 | 2.059 | **3.942** |
| Al(II)-O7 | 1.969 | 1.825 | 1.881 | **3.614** | **2.211** | 1.850 | 1.899 | **3.400** | **3.624** |
| Al(II)-O8 | 1.930 | 1.890 | 1.895 | 1.882 | 1.975 | 1.926 | 1.913 | 1.874 | 1.864 |
| Al(II)-O5 | 2.004 | **3.563** | 1.982 | 1.882 | 1.934 | **3.140** | 1.967 | 1.895 | 1.873 |
| Al(II)-O6 | 2.026 | **3.311** | **2.719** | 1.885 | 2.051 | 2.085 | **2.556** | 1.882 | 1.913 |
| Al(I)-O5 | 1.906 | 1.954 | 1.930 | **2.010** | 1.959 | 1.871 | 1.931 | **1.988** | 1.980 |
| Al(I)-O6 | 1.888 | 1.829 | 1.975 | 1.893 | 1.879 | 2.008 | 1.937 | 1.884 | 1.894 |
| Al(V)-Al(II) | 3.047 | 2.940 | 2.970 | **3.440** | **3.591** | 3.259 | 2.997 | 3.413 | **4.532** |
| Al(II)-Al(I) | 3.109 | **4.600** | 3.423 | 3.014 | 3.105 | 3.670 | 3.345 | 3.006 | 2.999 |
| RMS (Å) |  | 0.961 | 0.248 | 0.541 | 0.434 | 0.571 | 0.188 | 0.474 | 0.948 |
| Overall RMS (Å) |  | 0.605 | | | | 0.609 | | | |
| *∆E_1_, ∆E_2_ |  | -1024 | -1010 | -965 | -902 | -366 | -275 | -394 | -261 |
| Average ∆E_1_, ∆E_2_ |  | -975 | | | | -324 | | | |

* Subindexes at ∆E correspond to the equations in section 4.1.

Table S4. Atomic distances of the optimized initial and attacked molecular clusters (Å), on the {010} edge face, with the first H^+^ on O5, O6, O7, and O8, and various possibilities for the second attached H^+^ (Figure 2C). RMS values between the initial and protonated structures, the overall RMS across different protonations for the series, and the energy balance of protonation (kJ/mol). Bold distances indicate the most changed distances from the initial ones. PRE are written here as ∆Ei according to Eqs. (1) – (6).

| **Atomic distances** | **1^st^ H^+^ on O5** | | | | **1^st^ H^+^ on O6** | | | |
| --- | --- | --- | --- | --- | --- | --- | --- | --- |
|  | Initial | Optimized | | | Initial | Optimized | | |
| 2nd H^+^ on |  | O6 | O7 | O8 |  | O5 | O7 | O8 |
| Al(V)-O7 | 3.717 | **4.024** | **4.007** | **4.008** | 2.026 | 2.028 | **3.717** | 1.977 |
| Al(V)-O8 | 1.903 | 1.936 | 1.906 | **2.853** | 2.056 | 1.974 | 1.924 | **3.780** |
| Al(II)-O7 | 1.825 | 1.835 | 2.034 | 1.830 | 1.881 | 2.032 | 2.002 | 1.944 |
| Al(II)-O8 | 1.890 | 1.887 | 1.919 | 1.948 | 1.895 | 1.839 | 1.896 | 2.064 |
| Al(II)-O5 | 3.563 | **3.787** | 3.321 | **3.622** | 1.890 | 2.02 | **3.951** | 1.975 |
| Al(II)-O6 | 3.311 | **3.460** | 2.060 | **3.625** | 2.719 | **4.029** | 2.04 | **3.079** |
| Al(I)-O5 | 1.954 | 1.941 | 1.870 | 1.977 | 1.930 | **3.886** | 1.844 | 2.131 |
| Al(I)-O6 | 1.829 | 2.135 | 2.192 | 1.854 | 1.892 | 1.931 | **3.851** | 1.879 |
| Al(V)-Al(II) | 2.940 | 2.947 | 3.060 | 3.256 | 2.970 | 2.680 | 3.015 | 3.434 |
| Al(II)-Al(I) | 4.600 | 4.991 | 3.815 | 4.858 | 3.423 | 5.622 | 5.528 | 3.7300 |
| RMS (Å) |  | 0.204 | 0.502 | 0.355 |  | 1.025 | 1.260 | 0.591 |
| Overall RMS (Å) |  | 0.374 | | |  | 0.998 | | |
| *∆E_3_ (1H^+^) |  | -712 | -788 | -702 |  | -852 | -719 | -852 |
| Average ∆E_3_ (1H^+^) |  |  | -734 |  |  |  | -807 |  |
| ∆E_5_ (2H^+^) |  | -1736 | -1812 | -1727 |  | -1861 | -1728 | -1861 |
| Average ∆E_5_ (2H^+^) |  |  | -1758 |  |  |  | -1817 |  |
| **Atomic distances** | **1^st^ H^+^ on O7** | | | | **1^st^ H^+^ on O8** | | | |
|  | Initial | Optimized | | | Initial | Optimized | | |
| 2nd H^+^ on |  | O5 | O6 | O8 |  | O5 | O6 | O7 |
| Al(V)-O7 | 2.06 | 2.101 | 2.049 | 1.980 | 1.909 | 1.928 | 1.896 | 1.986 |
| Al(V)-O8 | 2.045 | 2.084 | 2.052 | **3.948** | **3.181** | **3.501** | **3.665** | **4.387** |
| Al(II)-O7 | 4.468 | **3.974** | 3.670 | 4.406 | 2.211 | **3.436** | 1.934 | **4.402** |
| Al(II)-O8 | 1.882 | 1.86 | 1.875 | 1.892 | 1.975 | 1.861 | 2.001 | 1.934 |
| Al(II)-O5 | 1.882 | 1.913 | 1.897 | 1.850 | 1.934 | 1.888 | 2.035 | 1.838 |
| Al(II)-O6 | 1.835 | 1.954 | 2.015 | 1.867 | 2.051 | 1.938 | **3.939** | 1.890 |
| Al(I)-O5 | 2.010 | 3.**036** | 1.995 | 2.040 | 1.959 | **2.885** | **3.822** | 2.017 |
| Al(I)-O6 | 1.893 | 1.895 | 2.094 | 1.914 | 1.879 | 1.899 | 1.845 | 1.915 |
| Al(V)-Al(II) | 3.440 | 3.458 | 3.432 | **4.654** | 3.591 | 4.684 | 3.005 | 4.828 |
| Al(II)-Al(I) | 3.014 | 3.458 | 3.190 | 2.975 | 3.105 | 3.407 | 5.515 | 2.967 |
| RMS (Å) |  | 0.389 | 0.272 | 0.715 |  | 0.614 | 1.162 | 0.886 |
| Overall RMS (Å) |  | 0.495 | | |  |  | 0.915 |  |
| *∆E_3_ (1H^+^) |  | -802 | -756 | -959 |  | -1056 | -895 | -778 |
| Average ∆E_3_ (1H^+^) |  |  | -839 |  |  |  | -910 |  |
| ∆E_5_ (2H^+^) |  | -1768 | -1721 | -1924 |  | -1958 | -1796 | -1680 |
| Average ∆E_5_ (2H^+^) |  |  | -1804 |  |  |  | -1811 |  |

* Subindexes at ∆E correspond to the equations in section 4.1.

Table S5. Atomic distances of the optimized initial and attacked molecular clusters (Å), from calculations performed on the {010} edge face, with H_3_O^+^ placed on O5, O6, O7, and O8, and various possibilities for the second attached H^+^ (Figure 2C). RMS values between the initial and protonated structures, the overall RMS between different protonation for the series, and the energy balance of protonation (kJ/mol) (Equations 4 and 6). Bold distances indicate the most changed distances from the initial ones. PRE are written here as ∆Ei according to Eqs. (1) – (6).

| **Atomic distances** | **H_3_O^+^ on O5** | | | | **H_3_O^+^ on O6** | | | |
| --- | --- | --- | --- | --- | --- | --- | --- | --- |
|  | Initial | Optimized | | | Initial | Optimized | | |
| 2nd H^+^ on |  | O6 | O7 | O8 |  | O5 | O7 | O8 |
| Al(V)-O7 | 3.274 | 3.87 | 3.635 | 4.606 | 2.023 | 1.891 | 3.291 | 1.905 |
| Al(V)-O8 | 1.937 | 1.946 | 1.991 | 3.332 | 2.033 | 2.045 | 2.036 | 3.746 |
| Al(II)-O7 | 1.85 | 1.854 | 1.975 | 1.735 | 1.899 | 3.028 | 1.965 | 1.889 |
| Al(II)-O8 | 1.926 | 1.881 | 1.907 | 1.979 | 1.916 | 1.849 | 2.128 | 1.897 |
| Al(II)-O5 | 3.14 | 3.658 | 3.187 | 2.755 | 1.967 | 1.902 | 2.059 | 3.52 |
| Al(II)-O6 | 2.085 | 3.685 | 2.079 | 1.908 | 2.556 | 4.463 | 2.165 | 3.759 |
| Al(I)-O5 | 1.871 | 1.964 | 1.887 | 1.896 | 1.931 | 4.724 | 1.876 | 1.873 |
| Al(I)-O6 | 2.008 | 1.972 | 2.02 | 2.012 | 1.937 | 1.942 | 2.107 | 2.001 |
| Al(V)-Al(II) | 3.259 | 2.932 | 3.355 | 4.901 | 2.997 | 2.869 | 3.61 | 2.919 |
| Al(II)-Al(I) | 3.423 | 4.933 | 3.702 | 3.4 | 3.345 | 6.081 | 3.29 | 4.938 |
| RMS (Å) |  | 0.747 | 0.155 | 0.813 |  | 1.423 | 0.472 | 0.967 |
| Overall RMS (Å) |  | 0.644 | | |  | 1.030 | | |
| *∆E_4_ (1H^+^) |  | -766 | -764 | -667 |  | -821 | -807 | -937 |
| Average ∆E_4_ (1H^+^) |  |  | -733 |  |  |  | -855 |  |
| ∆E_6_ (H_3_O^+^+1H^+^) |  | -1132 | -1130 | -1033 |  | -1096 | -1082 | -1213 |
| Average ∆E_6_ (H_3_O^+^+1H^+^) |  |  | -1098 |  |  |  | -1130 |  |
| **Atomic distances** | **H_3_O^+^ on O7** | | | | **H_3_O^+^ on O8** | | | |
|  | Initial | Optimized | | | Initial | Optimized | | |
| 2nd H^+^ on |  | O5 | O6 | O8 |  | O5 | O6 | O7 |
| Al(V)-O7 | 1.993 | 2.093 | 2.013 | 2.003 | 1.808 | 1.87 | 1.894 | 1.950 |
| Al(V)-O8 | 2.059 | 2.050 | 2.050 | **3.997** | 3.942 | **4.106** | **4.163** | **3.946** |
| Al(II)-O7 | 3.400 | 3.807 | **3.233** | **4.087** | 3.624 | **3.952** | **3.704** | **3.945** |
| Al(II)-O8 | 1.874 | 1.878 | 1.863 | 1.873 | 1.864 | 1.878 | 1.823 | 1.862 |
| Al(II)-O5 | 1.895 | 1.879 | 1.9 | 1.862 | 1.873 | 1.898 | 1.857 | 1.866 |
| Al(II)-O6 | 1.892 | 1.918 | 2.012 | 1.902 | 1.918 | 1.974 | 2.216 | 1.897 |
| Al(I)-O5 | 1.968 | **3.346** | 1.978 | 2.004 | 1.98 | 3.316 | 2.026 | 1.975 |
| Al(I)-O6 | 1.884 | 1.849 | 2.048 | 1.905 | 1.894 | 1.895 | 2.054 | 1.902 |
| Al(V)-Al(II) | 3.413 | 3.424 | 3.356 | 4.688 | 4.532 | 4.652 | 4.593 | 4.875 |
| Al(II)-Al(I) | 3.006 | 3.483 | 3.137 | 3.007 | 2.999 | 3.421 | 3.269 | 3.017 |
| RMS (Å) |  | 0.480 | 0.095 | 0.765 |  | 0.460 | 0.161 | 0.156 |
| Overall RMS (Å) |  | 0.524 | | |  | 0.295 | | |
| *∆E_4_ (1H^+^) |  | -786 | -780 | -686 |  | -781 | -856 | -922 |
| Average ∆E_4_ (1H^+^) |  |  | -751 |  |  |  | -853 |  |
| ∆E_6_ (H_3_O^+^+1H^+^) |  | -1180 | -1175 | -1080 |  | -1042 | -1117 | -1184 |
| Average ∆E_6_ (H_3_O^+^+1H^+^) |  |  | -1145 |  |  |  | -1114 |  |

* Subindexes at ∆E correspond to the equations in section 4.1.

Table S6. Atomic distances in the optimized initial and attacked molecular clusters (Å), on the {110} (Figure 3A) edge face, with H^+^ and H_3_O^+^ placed on O9, O10, and O11. RMS values between the initial and protonated structures, the overall RMS between different protonations for the series, and the energy balance of protonation (kJ/mol). Bold distances indicate the most changed distances from the initial ones. PRE are written here as ∆Ei according to Eqs. (1) – (6).

| **Atomic distances** |  | **H^+^** | | | **H_3_O^+^** | | |
| --- | --- | --- | --- | --- | --- | --- | --- |
|  | Initial | Optimized | | | Optimized | | |
|  |  | on O9 | on O10 | on O11 | on O9 | on O10 | on O11 |
| Al(VIII)-O9 | 1.824 | **2.036** | 1.823 | 1.779 | 1.890 | 1.890 | 1.877 |
| Al(VIII)-O11 | 1.945 | 1.920 | 1.981 | 1.910 | 1.940 | 1.940 | 1.954 |
| Al(IX)-O11 | 1.998 | 1.976 | 1.948 | **2.001** | 1.979 | 1.979 | 1.972 |
| Al(X)-O10 | 1.809 | 1.807 | **2.016** | **2.010** | **1.940** | **1.940** | **1.953** |
| Al(VIII)-Al(IX) | 3.033 | 3.030 | 3.053 | 3.045 | 3.021 | 3.021 | 3.022 |
| Al(IX)-Al(X) | 3.020 | 3.040 | 3.001 | 3.011 | 3.026 | 3.026 | 3.012 |
| RMS (Å) |  | 0.088 | 0.089 | 0.086 | 0.061 | 0.061 | 0.064 |
| Overall RMS (Å) |  | 0.087 | | | 0.062 | | |
| *∆E_1_, ∆E_2_ |  | -1102 | -1167 | -1134 | -479 | -479 | -493 |
| Average ∆E_1_, ∆E_2_ |  |  | -1134 |  |  | -484 |  |

* Subindexes at ∆E correspond to the equations in section 4.1.

Table S7. Atomic distances of the optimized initial and attacked molecular clusters (Å), on the {110} (Figure 3A) edge faces by placing two H^+^ and two H_3_O^+^ on O10 and O11. RMS between the initial and protonated structures, and the energy balance of the protonation (kJ/mol). Bold distances indicate the most changed distances from the initial ones. PRE is written here as ∆E according to Eqs. (1) – (6).

| **Atomic distances** |  | **2 H^+^** | **2 H_3_O^+^** |
| --- | --- | --- | --- |
|  | Initial | Optimized | Optimized |
| Al(VIII)-O9 | 1.824 | 1.763 | 1.823 |
| Al(VIII)-O11 | 1.945 | 2.122 | 1.923 |
| Al(IX)-O11 | 1.998 | **2.458** | 1.986 |
| Al(X)-O10 | 1.809 | 2.068 | **1.972** |
| Al(X)-O12 | 1.868 | 1.858 | **2.076** |
| Al(VIII)-Al(IX) | 3.033 | **3.304** | 3.056 |
| Al(IX)-Al(X) | 3.020 | 3.009 | 3.052 |
| RMS (Å) |  | 0.235 | 0.101 |
| ∆E |  | -1904 | -726 |

Table S8. Atomic distances of the optimized initial and attacked molecular clusters (Å), on the {130} (Figure 3C) edge face by placing one H^+^ or one H_3_O^+^ on O13. RMS values between the initial and protonated structures, and the energy balance of protonation (kJ/mol) (Equation 1). Bold distances indicate the most changed distances from the initial ones. PRE are written here as ∆E_i_ according to Eqs. (1) – (6).

| **Atomic distances** |  | **H^+^** | **H_3_O^+^** |
| --- | --- | --- | --- |
|  | Initial | Optimized | Optimized |
|  |  | O13 | O13 |
| Al(XI)-O13 | 2.010 | **2.707** | **3.353** |
| Al(XI)-O*c* | 1.926 | 1.927 | 1.960 |
| Al(XII)-O13 | 1.940 | 2.198 | 2.107 |
| Al(XII)-O*c* | 1.929 | 1.952 | 2.023 |
| Al(XI)-Al(XII) | 3.008 | 3.294 | 3.528 |
| RMS (Å) |  | 0.356 | 0.650 |
| ∆E_1_ |  | -930 | -297 |

* Subindexes at ∆E correspond to the equations in section 4.1.
